# Memantine Attenuates Alzheimer’s Disease-Like Pathology and Cognitive Impairment

**DOI:** 10.1371/journal.pone.0145441

**Published:** 2015-12-23

**Authors:** Xiaochuan Wang, Julie Blanchard, Inge Grundke-Iqbal, Khalid Iqbal

**Affiliations:** 1 Department of Neurochemistry, Inge Grundke-Iqbal Research Floor, New York State Institute for Basic Research in Developmental Disabilities, Staten Island, New York, United States of America; 2 Department of Pathophysiology, Key Laboratory of Neurological Disease of National Education Ministry, Tongji Medical College, Huazhong University of Science and Technology, Wuhan, Hubei, China; Centre Hospitalier de l'Université Laval, CANADA

## Abstract

Deficiency of protein phosphatase-2A is a key event in Alzheimer’s disease. An endogenous inhibitor of protein phosphatase-2A, inhibitor-1, I_1_^PP2A^, which inhibits the phosphatase activity by interacting with its catalytic subunit protein phosphatase-2Ac, is known to be upregulated in Alzheimer’s disease brain. In the present study, we overexpressed I_1_^PP2A^ by intracerebroventricular injection with adeno-associated virus vector-1-I_1_^PP2A^ in Wistar rats. The I_1_^PP2A^ rats showed a decrease in brain protein phosphatase-2A activity, abnormal hyperphosphorylation of tau, neurodegeneration, an increase in the level of activated glycogen synthase kinase-3beta, enhanced expression of intraneuronal amyloid-beta and spatial reference memory deficit; littermates treated identically but with vector only, i.e., adeno-associated virus vector-1-enhanced GFP, served as a control. Treatment with memantine, a noncompetitive NMDA receptor antagonist which is an approved drug for treatment of Alzheimer’s disease, rescued protein phosphatase-2A activity by decreasing its demethylation at Leu309 selectively and attenuated Alzheimer’s disease-like pathology and cognitive impairment in adeno-associated virus vector-1-I_1_^PP2A^ rats. These findings provide new clues into the possible mechanism of the beneficial therapeutic effect of memantine in Alzheimer’s disease patients.

## Introduction

Alzheimer’s disease (AD) is one of the most common neurodegenerative disorders in the elderly population, associated with progressive memory loss and cognitive impairment (www.nia.nih.gov; www.alz.org). It is characterized by the presence of two hallmark lesions: extracellular senile plaques and intracellular neurofibrillary tangles. The former consists of β-amyloid [1]. Neurofibrillary tangles are made up of paired helical filaments (PHFs). The major protein subunit of PHFs is the microtubule-associated protein tau in an abnormally hyperphosphorylated state [2, 3]. Although the induction of AD pathology in transgenic animal models induces cognitive impairment, mechanisms of AD involving tau and Aβ pathologies remains to be established. Protein phosphatase-2A (PP2A), which accounts for ~70% of tau phosphatase in human brain [4], is compromised in AD brain [5, 6]. PP2A activity is regulated by two endogenous inhibitors of PP2A, inhibitor-1 (I_1_^PP2A^) and inhibitor-2 (I_2_^PP2A^)[7, 8]. Both mRNA and protein expressions of I_1_^PP2A^ and I_2_^PP2A^ are increased in AD brain [9, 10]. I_1_^PP2A^ is involved in some important physiological events, such as cell proliferation, apoptosis, mRNA transport, and transcription [11]. I_1_^PP2A^ only interacts with the PP2A catalytic subunit PP2Ac and there is no interaction between I_1_^PP2A^ and PP2A A or B regulatory subunit. The minimal region required for the association with PP2Ac as well as PP2A inhibition is localized at N-terminal isotype specific containing region of I_1_^PP2A^ [8]. Previously we showed that memantine can rescue PP2A activity deficit in rat hippocampal slices in culture [12].

Memantine is approved for treatment of moderate to severe AD [13, 14]. As a noncompetitive glutamatergic NMDA receptor antagonist, memantine can protect neurons from Aβ-induced glutamate-mediated toxicity by attenuating phosphorylation of tau through a decrease in glycogen synthase kinase-3beta (GSK-3β) activity via the PI-3/Akt kinase-dependent pathway [15, 16]. Memantine was also shown to reduce the levels of secreted APP and Aβ both in human neuroblastoma SK-N-SH cells and in neuronal cultures and APP/PSI transgenic mice [17, 18]. Previously, we showed that memantine can rescue the decrease in PP2A activity induced by I_2_^PP2A^ and inhibit the Alzheimer’s type abnormal hyperphosphorylation of tau and associated neurodegeneration [12, 19]. However, the exact mechanism of regulation of PP2A activity by memantine is still vague. In the present study, we orally administered memantine to rats intracerebroventricularly infected with adeno-associated virus vector-1 (AAV1)-I_1_^PP2A^ and as control with AAV1-GFP for 3 months starting at the age of 45 days. Memantine rescued PP2A activity via decreasing demethylation of PP2A at Leu309 selectively in AAV1-I_1_^PP2A^ rat brain, attenuated tau hyperphosphorylation and neurodegeneration. Attenuation of spatial learning and memory impairment in memantine-treated animals was associated with a reversal in I_1_^PP2A^-induced decrease in pSer-133 CREB. These findings suggest that memantine may also delay and prevent AD pathology by restoring PP2A activity.

## Materials and Methods

### Generation of pTRUE12-I_1_^PP2A^ and vector packing

Employing pEGFP-N3/ I_1_^PP2A^ (wt) generated by us previously (6) as a template, I_1_^PP2A^ cDNA was obtained by PCR with primer 1 (5’-GGCACTAGTATGGAGATGG GCAGAC) and primer 2 (5’-TGCGATATCTTAGTCATCATCTTCTCCCTC). The SpeI site underlined in the primer 1 and the EcoR V site underlined in the primer 2 were used to clone the fragment into pcDNA3.1 vector (Invitrogen, Carlsbad, CA, USA). The plasmid was verified by DNA sequencing. The I_1_^PP2A^ cDNA fragment was then cloned into the multicloning site of the AAV viral genome containing plasmid pTRUF12 and expression was driven by the CMV promoter/enhancer. Recombinant AAV serotype 1 virus was generated and titers were calculated from standard curve generated from pTRUF as previously described [20–22].

GFP was expressed as a reporter protein because the AAV1’s pTRUF12 vector contains a GFP sequence after the Kozak consensus sequence which plays a major role in the initiation of the translation process.

### Intracerebroventricular injection of AAV

On the day of birth, designated as P 0.5, rat pups were individually anesthetized on ice, and 2 μl of AAV1-I_1_^PP2A^ was injected into each lateral ventricle with a specially designed fine 10 μl Hamilton syringe with a 30 gauge/0.5 inch hypodermic cemented needle (Hamilton Syringe Col, Reno, NV, USA). A total of 2.73×10^10^ AAV1 genomic equivalents in 4 μl were injected into each rat brain. Control animals were treated identically except with vector only, i.e., AAV1-GFP.

### Treatment of animals with Memantine

After 3 weeks pups were weaned and only male animals were employed in the present study. We divided the littermates into two groups, one group for memantine treatment and another for vehicle control (H_2_O). Meanwhile, we coded each pup and each cage for the following experiments performed by ourselves but it was not a double blind study. After 1½ months, 9 AAV1-GFP- and 7 AAV1-I_1_^PP2A^-infected animals were administered *per os* memantine (daily *per os* 2 mg/kg, 6 days/week; Cat#298080010, Acros Organics), 9 AAV1-GFP- and 6 AAV1-I_1_^PP2A^-infected animals received water as vehicle control for 12 weeks. After 12 weeks of chronic treatment with memantine, animals were submitted to a general behavioral battery, followed by cognitive tests.

### Animal housing

Normal Wistar rats were purchased from Charles River Laboratories (Germantown, MD, USA) and bred and maintained at the Animal Colony of the New York State Institute for Basic Research according to the PHS Policy on Human Care and Use of Laboratory Animals (revised March 15, 2010). Animals were housed in an animal room facility maintained at 23°C with a light-dark cycle of 12 h (lights off at 6:00 p.m.) and with accessible food and water *ad libitum*. All procedures carried out on animals were conducted in strict compliance with protocols approved by the Institutional Animal Care and Use Committee (IACUC) of the New York State Institute for Basic Research.

### Western blot analysis

For Western blot analysis, to prevent any effect of anesthesia on tau phosphorylation rats were sacrificed by cervical dislocation and their brains were extracted directly in the ice-cold homogenizing buffer. Ventricular area, cerebral cortex, and hippocampus, were quickly dissected out and homogenized on ice to generate 12% (w/v) homogenate in buffer containing 50 mM Tris HCl (pH 7.4), 8.5% sucrose, 2 mM EDTA, 10 mM β-mercaptoethanol, 0.2 mM phenylmethylsulfonylfluoride, 10 μg/ml leupeptin, and 2 μg/ml each of aprotinin and pepstatin A.

Western blots were carried out as described previously [22]. The antibodies employed in the present study are listed as follows:

The primary antibodies used were purified mouse anti-PP2A catalytic α (1:10000; BD Transduction Laboratories^™^), demethylated anti-PP2AC 4b7 (1:5000; Millipore), pan-tau antibody, rabbit polyclonal antibody (pAb) 92e (1:5000; [23]), phosphospecific tau antibodies: Tau pAb pS199 (1:1000, BioSource, Camarillo, CA, USA), Tau pAb pT205 (1:1000; BioSource), Tau pAb pS214 (1:500; BioSource), Tau monoclonal antibody (mAb) M4 to phosphorylated Thr 231/Ser-235 (1:1500; [24]), Tau mAb 12E8 to phosphorylated Ser 262/356 (1:500; [25]), Tau pAb pS396 (1:1000; BioSource), Tau pAb R145 to pS422 (1:3000; [26]), I_1_^PP2A^ mAb (5G6, 8.4 μg/ml; generated against recombinant human I_1_^PP2A^; unpublished), mAb GFP (1:1000; Rockland, Gilbertville, PA, USA), mAb β-actin (1:2000; Sigma), mAb DM1A to α-tubulin (1:1000; Sigma), and rabbit pAb βIII tubulin (1:5000; Covance, Princeton, NJ, USA). The rabbit monoclonal antibodies used for the Western blots were rabbit mAb phospho-CREB 87G3 to pSer133 (1:1000; Cell Signaling, Danvers, MA, USA), rabbit mAb 48H2 to CREB (1:1000; Cell Signaling), rabbit mAb Phospho-GSK-3β to pSer9 (1:1000; Cell Signaling), and rabbit mAb 27C10 to GSK-3β (1:2000; Cell Signaling). Immunoreactive protein bands were visualized with ECL reagents (Pierce, Rockford, IL, USA). Western blot analysis employed 3 animals/group.

### PP2A activity assay

PP2A activity was assayed by ELISA in rat hippocampus homogenate [22]. Briefly, 96 well plates were coated at room temperature for 8 h with a 17 amino acid phosphopeptide corresponding to tau 194–207 to which 3 lysines were added at the C-terminus and Ser199 was phosphorylated in 35 mM NaHCO_3_ and 50 mM NaF. After blocking with a protein free blocking buffer (Pierce, Rockford, IL, USA) the enzymatic reaction was performed adding 5 μg of hippocampus homogenized in reaction buffer (50 mM Tris-HCl pH 7, 20 mM β-mercaptoethanol, 2 mM MnCl_2_, 0.1 mg/ml BSA) and incubating at 30°C for 30 min (in the presence or absence of 15 nM okadaic acid). The reaction was stopped by adding blocking buffer containing 50 mM NaF, followed by overnight incubation with Tau-1 antibody that recognizes tau unphosphorylated at Ser198/199/202 (1:25,000). After 1 h incubation with horseradish peroxidase (HRP)-conjugated secondary antibody (1:1200; Jackson ImmunoResearch, West Grove, PA, USA), the colorimetric reaction was performed using 75 μl of tetramethylbenzidine (TMB) (Sigma, St. Louis, MO, USA) and was followed for 16 min at 650 nm.

### Immunohistochemistry and double immunofluorescence staining

For immunohistochemical studies, rats anesthetized with chloral hydrate (30 mg/kg, intraperitoneally) were perfused through the aorta with 100 ml 0.9% NaCl followed by 100 ml phosphate buffer containing 4% paraformaldehyde. Brains were removed and postfixed in 4% paraformaldehyde overnight then equilibrated in a cryoprotectant solution of 30% sucrose/PBS and stored at 4°C.

Immunohistochemistry and immunofluorescence staining were carried out as described previously [22]. The antibodies employed in the present study are listed as follows:

The primary antibodies used were I_1_^PP2A^ mAb (5G6, 8.4 μg/ml), pAb Aβ34–40 (1:500; Invitrogen), pAb Aβ36–42 (1:500; Invitrogen), mAb GFP (1:500; Rockland), Tau mAb M4 to phosphorylated Thr 231/Ser-235 (1:1000; [24]), Tau mAb 12E8 to phosphorylated Ser 262/356 (1:250), mAb SMI52 to MAP2a,b (1: 1000; Covance), mAb synaptophysin (1:200; Chemicon, Temecula, CA, USA), and pAb synapsin-1 (1:500; Stressgen, Ann Arbor, MI, USA). Secondary antibodies used were: Alexa 488-conjugated goat anti-mouse antibody (1:1000) and Cy3 goat anti-rabbit antibody (1:1,000) (Jackson Immunoresearch, West Grove, PA). Finally, the sections were washed in TBS and mounted on glass slides with ProLong Gold antifade reagent (Invitrogen). Immunohistochemical analysis employed 3 animals/group.

### Image analysis and semiquantification of immunofluorescence

The sections were subjected to confocal microscopy (PCM 2000 Confocal Imaging System; Nikon, Melville, NY, USA) for quantitative analysis using a 40 objective. Area of interest was outlined, and maximum projection images were then generated based on confocal *z* stacks. The antibody staining was semiquantitated by measuring mean fluorescence intensities (MFIs) with NIH Image J software (U.S. National Institutes of Health, Bethesda, MD, USA). Six to ten images of sections stained for MAP2, synaptophysin, or synapsin were obtained per hippocampal CA1 and CA3 region, respectively. MFI per square micrometer area was calculated by dividing the MFI units by the area of outlined regions.

### Fluoro-Jade B labeling

Fluoro-Jade B labeling were carried out as described previously [22]. Briefly, tissue sections were mounted on 2% gelatin-coated slides and then dried at room temperature overnight. Slides were briefly rinsed in distilled water, followed by 3-min incubation in 100% alcohol, 1 min in 70% alcohol, 1 min in 30% alcohol, and a 1-min rinse in distilled water. The sections were then treated with a solution of 0.06% potassium permanganate for 15 min with gentle shaking and rinsed in distilled water for 1 min. The staining solution was prepared to contain a 0.001% Fluoro-Jade B (Chemicon) in 0.1% acetic acid. After incubating for 30 min in the staining solution, the sections were rinsed for 1 min in each of three distilled water washes and dried. The dry sections were washed 3 times in xylene 2 min each before mounting with DPX (Fluka, Milwaukee, WI, USA). Images were captured using a Nikon 90i microscope equipped with epifluorescence optics.

### Behavioral procedures

#### Neurological examination

A battery of behavioral tests were carried out to perform a quantitative evaluation of anxiety status, exploratory activity, reflexes, muscle strength and function and motor coordination. We first assessed anxiety and exploratory activity of rats using an open-field free-exploration test. Then, using a scoring system adapted from Korenova et al. [27], we were able to measure the consequences of the neurodegenerative processes on neurological and neuromuscular functions (Table 1).

**Table 1:** Neuroscale scoring protocol (adapted from Korenova et al., 2009).

*General observation (normal 0; maximum 4 points)*		4
1	Abnormal posture	
1	Forelimb paralysis	
1	Hind-limb paralysis	
1	Vocalization during examination	
*Hind-limb extension reflex (normal 0; maximum 3 points)*		3
0	Hind-limb extended with spreading toes	
1	Hind-limb extended with spreading toes, distance between limbs is shorter than in previous scoring	
2	Hind-limb almost in contact, toes spread (or flexed)	
3	Hind-limb extended clasped or crossed with toes flexed	
*Beam walking test—escape latency– 3 testing conditions (normal 0–5; maximum 15 points)*		15
0	Latency ≤2s	
1	Latency 2.1–4.0s	
2	Latency 4.1–6.0s	
3	Latency 6.1–10.0s	
4	Latency 10.1s and more	
5	Animal not able to perform the task	
*Beam walking test—Number of hind-limb slips– 3 testing conditions (normal 0–5; maximum 15 points)*		15
0	Hind-limb slips ≤1	
1	Hind-limb slips 1.1–2.0	
2	Hind-limb slips 2.1–3.0	
3	Hind-limb slips 3.1–5.0	
4	Hind-limb slips5.1–8.0	
5	More than 8 hind-limb slips; animal is not able to perform the task	
*Prehensile traction-test—latency to fall (normal 0; maximum 5 points)*		5
5	Latency ≤2s	
4	Latency 2.1–4.0s	
3	Latency 4.1–6.0s	
2	Latency 6.1–10.0s	
1	Latency 10.1–15.0s	
0	More than 15s	
***Maximum points***		***42***

#### Open-field free-exploration

Anxiety and exploratory activity were evaluated allowing rats to freely explore an open field for 20 min. The testing apparatus was a classic open field (i.e., a PVC square arena of 100 x 100 cm, with 70 cm high walls). The open field was placed in a part of the room separated from the experimentator and the control station with a black opaque curtain. Rats were individually submitted to a single 20 min-session. Because for rodents the middle of an unfamiliar arena is anxiogenic, anxiety was studied analyzing the percentage of time spent in the middle of the arena. To assess exploratory activity, the total distance the animals covered in the arena was tracked and measured. Data collection was performed using tracking files of the experiment recorded with SMART (Pan Lab/San Diego Instruments) version 2.0.14 software.

#### Beam walking test

For the beam-walking test, 3 sorts of traversing segments were used (3 cm x 3 cm, 4 cm x 2 cm (traversing segment is 2 cm) and a round beam of 3.5 cm diameter). All had the same length of 200 cm and were placed 75 cm above the floor. Two training and two testing trials were performed. Traversing latency and number of hind-limb slips made during test performance were determined and scored according to the pre-defined rating scale (Table 1). The task was repeated at different sensitivity according to the following scheme:beam with a square section of 3 cm x 3 cm (first day); beam with a rectangular cross-section of 4 cm x 2 cm (second day); beam with a round cross section of 3.5 cm diameter (third day).

#### Prehensile traction test

The rats were allowed to grasp with its forepaws a horizontal steel wire (3 mm in diameter) suspended 75 cm above a padded surface. Latency to fall from the wire was measured. The scoring conditions are notified in Table 1.

### Cognitive test

#### Water-maze spatial reference memory task

Spatial reference learning and memory was evaluated in the water maze using a procedure adapted from that described previously [28]. The test requires that rats use a spatial navigational strategy based on a spatial representation of the environment to find a fixed submerged escape platform. The procedure was performed in a 180 cm diameter circular tank. The pool was filled with water (21°C ± 1) made opaque by adding white non-toxic paint. Acquisition started with the escape platform (13 cm diameter submerged 1 cm below water surface) in the Northwest quadrant and each animal was given 90 s to find the platform. If the rat did not find the platform in 90 s, it was gently guided to it. At the end of each trial the rat was left on the platform for 20 s then dried and returned to its home cage until the next trial. Three such acquisition trials were given on each day for three consecutive days. The measures of learning were the time and the distance swum to reach the escape platform. Rat behavior in the water-maze was monitored by a Samsung Digital Camera (SDC 4304) mounted to the ceiling and tracked and timed by a SMART (Pan Lab/San Diego Instruments) version 2.0.14 software.

### Statistical Analyses

Statistical analyses were performed using SPSS 18.0 statistical software. The one-way ANOVA procedure, followed by least-square differences post-hoc tests were used to determine the statistical significance of differences of the means. For a single comparison, the significance of differences between means was determined by the t test. Two-way ANOVAs were performed for data on exploration in the open field and training in the water maze. Kruskal-Wallis U-test was used to perform analysis of the neuroscore. Statistical significance was accepted at the 95% confidence level (p<0.05). Data are expressed as means ± SE.

## Results

### I_1_^PP2A^ is upregulated in AAV1-I_1_^PP2A^ rats

**Fig 1:**
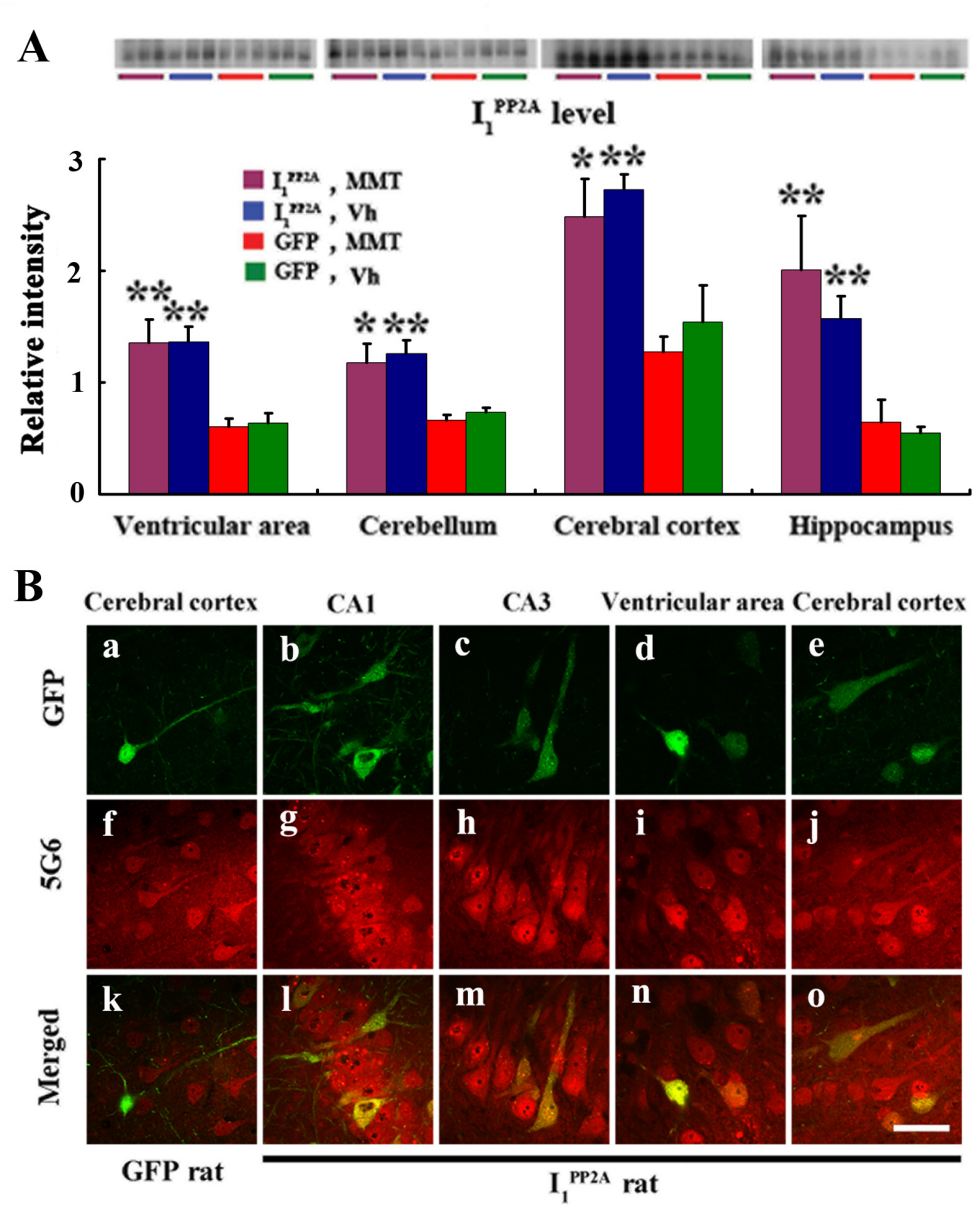
Infection with AAV1-I_1_^PP2A^ upregulates I_1_^PP2A^ in rat brain. A) Four and one-half months post-infection, Western blots developed with mouse monoclonal anti-I_1_^PP2A^ (5G6) showed a significant increase in the level of the transgene (normalized with actin as loading control; actin blot the same as shown in Fig 4A) in the ventricular area, the cerebellum, the cerebral cortex and the hippocampus in AAV1-I_1_^PP2A^ rats as compared with that in AAV1-GFP control rats. Memantine treatment didn’t alter the level of I_1_^PP2A^ in either AAV1-I_1_^PP2A^ or AAV1-GFP rats. *P<0.05, **P<0.01 vs. AAV1-GFP control rats. B) Immunofluorescence staining with anti-GFP showed transgene expression widely spread in the cerebral cortex(a and e), the hippocampus (b and c) and the ventricular area (d) in AAV infected rats. I_1_^PP2A^ including the endogenous protein was observed with 5G6 staining both in AAV1-GFP rats (f) and AAV1-I_1_^PP2A^ rats (g-j). The double staining showed that GFP colocalized with 5G6 only in AAV1-I_1_^PP2A^ rats (l-o), not in AAV1-GFP rats (k). 3–4 rats were employed in each experiment. MMT, memantine; Vh, vehicle. Scale bar, 50 μm.

Our previous studies showed efficient expression of a transgene in the brain by ICV infection using AAV1 delivery system in newborn rats [22, 29]. In order to study the effect of I_1_^PP2A^ in AD-like pathology we infected the brains of rat pups within 24 hours of birth. P 0.5 Wistar rats were bilaterally injected with AAV1 encoding I_1_^PP2A^ and GFP or, as control, AAV1-GFP alone into the cerebral lateral ventricles. Western blots and immunofluorescence staining were employed to detect and evaluate the expression and distribution of the virus encoded I_1_^PP2A^ transgene. Using 5G6, a specific monoclonal antibody against I_1_^PP2A^, Western blots results showed that the level of I_1_^PP2A^ from the site of injection, namely the ventricular area, to the cerebellum, the cerebral cortex and the hippocampus in AAV1-I_1_^PP2A^ rats, was significantly increased when compared with that in AAV1-GFP control rats. Memantine treatment didn’t influence the level of I_1_^PP2A^ in either AAV1-I_1_^PP2A^ rats or AAV1-GFP rats (Fig 1A). Immunofluorescence staining with anti-GFP also showed transgene delivery widely spread in AAV-treated rats, especially in the cerebral cortex (Fig 1Ba and 1Be), the hippocampus (Fig 1Bb and 1Bc) and the ventricular area (Fig 1Bd). Moreover, I_1_^PP2A^ including the endogenous protein was observed with 5G6 staining both in AAV1-GFP rats (Fig 1Bf) and AAV1-I_1_^PP2A^ rats (Fig 1Bg–1Bj). However, the double staining showed that GFP colocalized with 5G6 only in AAV1-I_1_^PP2A^ rats (Fig 1Bl–1Bo). These data suggested that I_1_^PP2A^ can be upregulated via AAV delivery system.

### Memantine rescues AAV1-I_1_^PP2A^-induced PP2A inhibition

**Fig 2:**
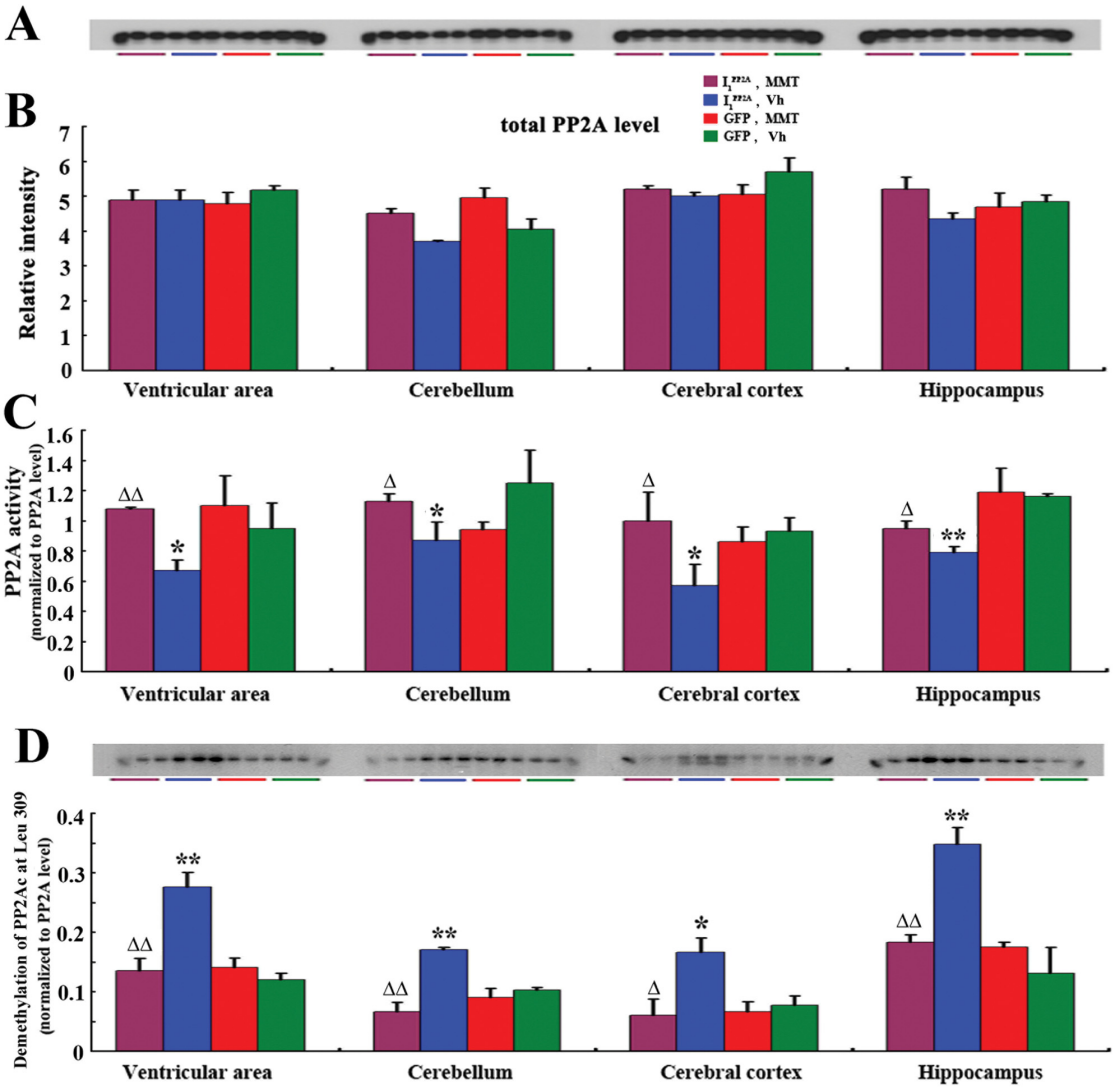
Memantine restores PP2A activity by decreasing the demethylation of PP2Ac at Leu 309. A, B) Western blots developed with mouse anti-PP2A catalytic α (1:10000; BD Transduction Laboratories^™^) showed no significant change in the level of PP2Ac in the AAV1-I_1_^PP2A^ rat brain when compared with AAV1-GFP control group and treatment with memantine didn’t influence the level of PP2Ac. C) PP2A activity as determined by phosphatase ELISA was markedly decreased in all brain areas studied in AAV1-I_1_^PP2A^ rats. Memantine only reversed pathologic change of PP2A activity in AAV1-I_1_^PP2A^ rats, but didn’t influence the level of PP2A activity in AAV1-GFP rats. *P<0.05, **P<0.01 AAV1-I_1_^PP2A^ vs. AAV1-GFP control rats; ^Δ^P<0.05, ^ΔΔ^P<0.01, AAV1-I_1_^PP2A^ with memantine vs. AAV1_1_^PP2A^ vehicle. D) Western blots developed with anti-demethylated PP2Ac (4b7) showed that the demethylation of PP2Ac at Leu 309 in AAV1-I_1_^PP2A^ rats with a chronic memantine treatment was markedly decreased as compared with vehicle-treated- I_1_^PP2A^ rats. Memantine did not influence the demethylation of PP2Ac in AAV1-GFP rats. 3–4 rats were employed in each experiment. *P<0.05, **P<0.01 AAV1-I_1_^PP2A^ vs. AAV1-GFP control rats; ^Δ^P<0.05, ^ΔΔ^P<0.01 AAV1-I_1_^PP2A^-memantine vs. AAV1-I_1_^PP2A^-Vh.

We previously reported that I_1_^PP2A^ regulated the PP2A activity by directly interacting with and inhibiting the activity of its catalytic subunit in NIH3T3 cells [8]. However, whether I_1_^PP2A^ inhibits PP2A activity in vivo was not known. In the present study, ELISA [22] and Western blots were carried out to detect PP2A activity in AAV1-I_1_^PP2A^ rats and AAV1-GFP rats. We found that there was no significant change in the level of PP2Ac in the AAV1-I_1_^PP2A^ rat brain when compared with AAV1-GFP control group (Fig 2A and 2B). However, the activity of PP2A was markedly decreased in all brain areas of AAV1-I_1_^PP2A^ rats studied (Fig 2C). These data suggested that upregulation of I_1_^PP2A^ induced inhibition of PP2A activity.

We previously reported that memantine could restore the OA-induced inhibition of PP2A in hippocampal slices and I_2_^PP2A^-induced inhibition of PP2A in PC12 cells [12, 19]. In the present study we investigated the effect of memantine on brain PP2A activity in AAV1-I_1_^PP2A^ rats. One and one half month old, 9 AAV1-GFP and 7 AAV1-I_1_^PP2A^ infected rats were administered *per os* with 2 mg/kg memantine, and 9 AAV1-GFP and 6 AAV1-I_1_^PP2A^ infected animals with water as vehicle control for 12 weeks (6 days per week). We found that only pathologic change of PP2A activity in AAV1-I_1_^PP2A^ rats was reversed; the normal level of PP2A activity in AAV1-GFP rats was not influenced by memantine (Fig 2C).

In order to learn how memantine reverses the inhibition of PP2A activity in I_1_^PP2A^ rats, we investigated the involvement of the demethylation of PP2Ac. As a non-competitive NMDA-receptor antagonist with antioxidative properties, memantine was previously reported to interact with P450 system and inhibit cytochrome P450 mediated monooxygenase functions [30]. Cytochrome P450 monooxygenase is known to possess the function of demethylation [31]. Therefore, in order to investigate the effect of memantine on possible demethylation of PP2Ac in AAV1-I_1_^PP2A^ rats, we examined the demethylation of PP2Ac in all rats studied using Western blots with anti-demethylated PP2Ac, 4b7. We found that the demethylation of PP2Ac at Leu 309 in AAV1-I_1_^PP2A^ rats with a chronic memantine treatment was markedly decreased when compared with vehicle-treated- I_1_^PP2A^ rats. Interestingly, memantine did not influence the demethylation of PP2Ac at Leu 309 in AAV1-GFP rats (Fig 2D). Taken together, these results revealed that upregulation of I_1_^PP2A^ induced a decrease in PP2A activity and that memantine could restore the PP2A activity to normal level by decreasing the demethylation of PP2Ac.

### *In vitro* inhibition of PP2A activity with I_1_^PP2A^ is reversed by memantine

**Fig 3:**
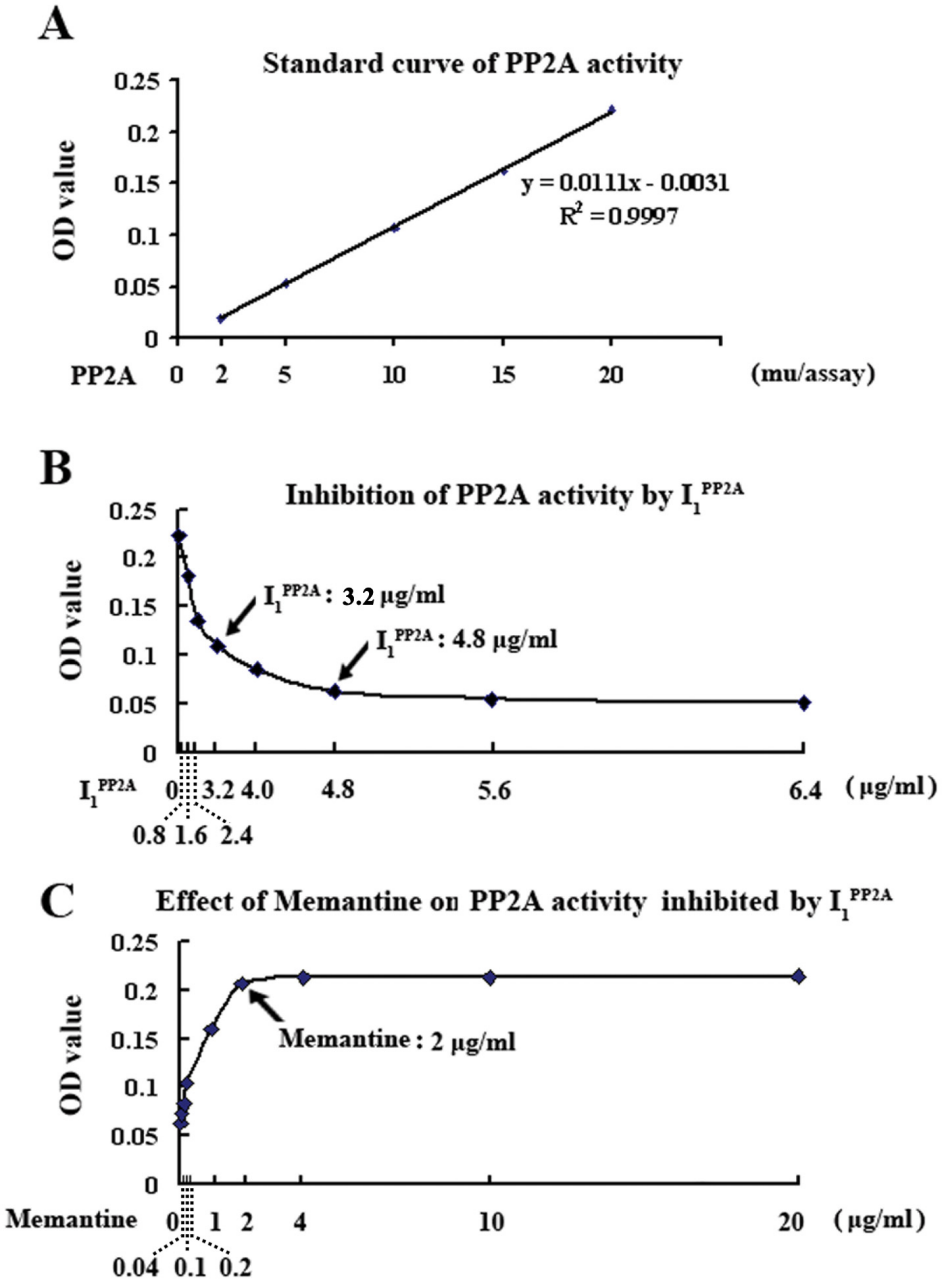
*In vitro* inhibition of PP2A activity with I_1_^PP2A^ is prevented by memantine. A) The standard curve of PP2A activity assay showing linearity of the reaction up to 20 mu PP2A/assay of purified PP2A holoenzyme in dephosphorylation of tau phosphopeptide P17. B) Recombinant I_1_^PP2A^ concentration-dependent inhibition of PP2A activity. C) 20 mu PP2A/assay purified PP2A and 4.8 μg/ml recombinant I_1_^PP2A^ determined from A and B were employed to evaluate the effect of memantine on inhibition of PP2A activity by I_1_^PP2A^. Memantine, 2 μg/ml assay, completely prevented the inhibition of PP2A with I_1_^PP2A^. 3–4 rats were employed in each experiment.

To learn whether memantine had any direct effect, we investigated its effect on inhibition of PP2A activity by I_1_^PP2A^ *in vitro*. First, we generated a standard curve of dephosphorylation of a synthetic a tau phosphopeptide p17 corresponding to tau_194–207_ with PP2A purified from bovine brain [32] at Tau-1 site [22]. The curve showed an O.D. change of ~0.2 at 20 mu PP2A/assay in the phosphatase ELISA (Fig 3A). Next, a concentration curve of inhibition of PP2A activity by recombinant I_1_^PP2A^ was generated. At 3.2 μg/ml and 4.8 μg/ml, I_1_^PP2A^ inhibited PP2A activity by 50.0% and 72.6%. Further increase in I_1_^PP2A^ had no significant increase in inhibition of PP2A activity (Fig 3B). Therefore, 20 mu/assay purified PP2A and 4.8 μg/ml recombinant I_1_^PP2A^ were employed to evaluate the effect of memantine on inhibition of PP2A activity by I_1_^PP2A^. We found that 2 μg/ml or high concentration memantine restored the decrease of PP2A activity induced by I_1_^PP2A^ to normal level *in vitro* (Fig 3C). Together with our previous report that showed that memantine did not have direct interaction with PP2A [12], these findings suggest that memantine directly interacts with I_1_^PP2A^, and blocks its interaction with PP2A.

### Memantine inhibits tau phosphorylation and neurodegeneration induced by I_1_^PP2A^

**Fig 4:**
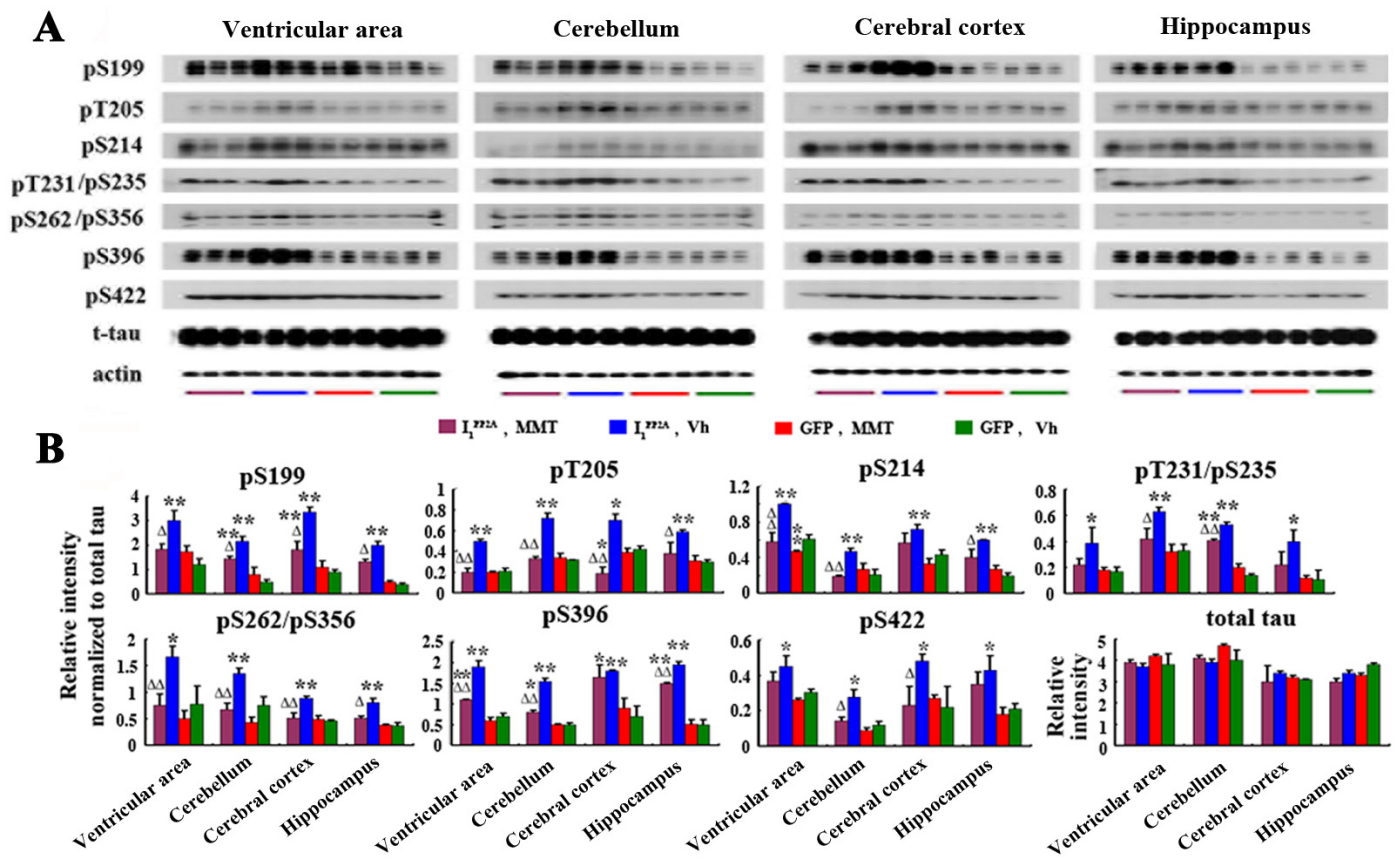
Memantine inhibits I_1_^PP2A^-induced tau hyperphosphorylation. A, B) Western blots showed increase in abnormal hyperphosphorylation of tau in AAV1-I_1_^PP2A^ rats. Quantitative analysis of the blots of hyperphosphorylated tau normalized with total tau showed significant increase in tau hyperphosphorylation at several sites studied and memantine was found to reverse this change. *P<0.05, **P<0.01 AAV1-I_1_^PP2A^ vs. AAV1-GFP rats; ^Δ^P<0.05, ^ΔΔ^P<0.01 AAV1-I_1_^PP2A^-memantine vs. AAV1-I_1_^PP2A^-vehicle.

**Fig 5:**
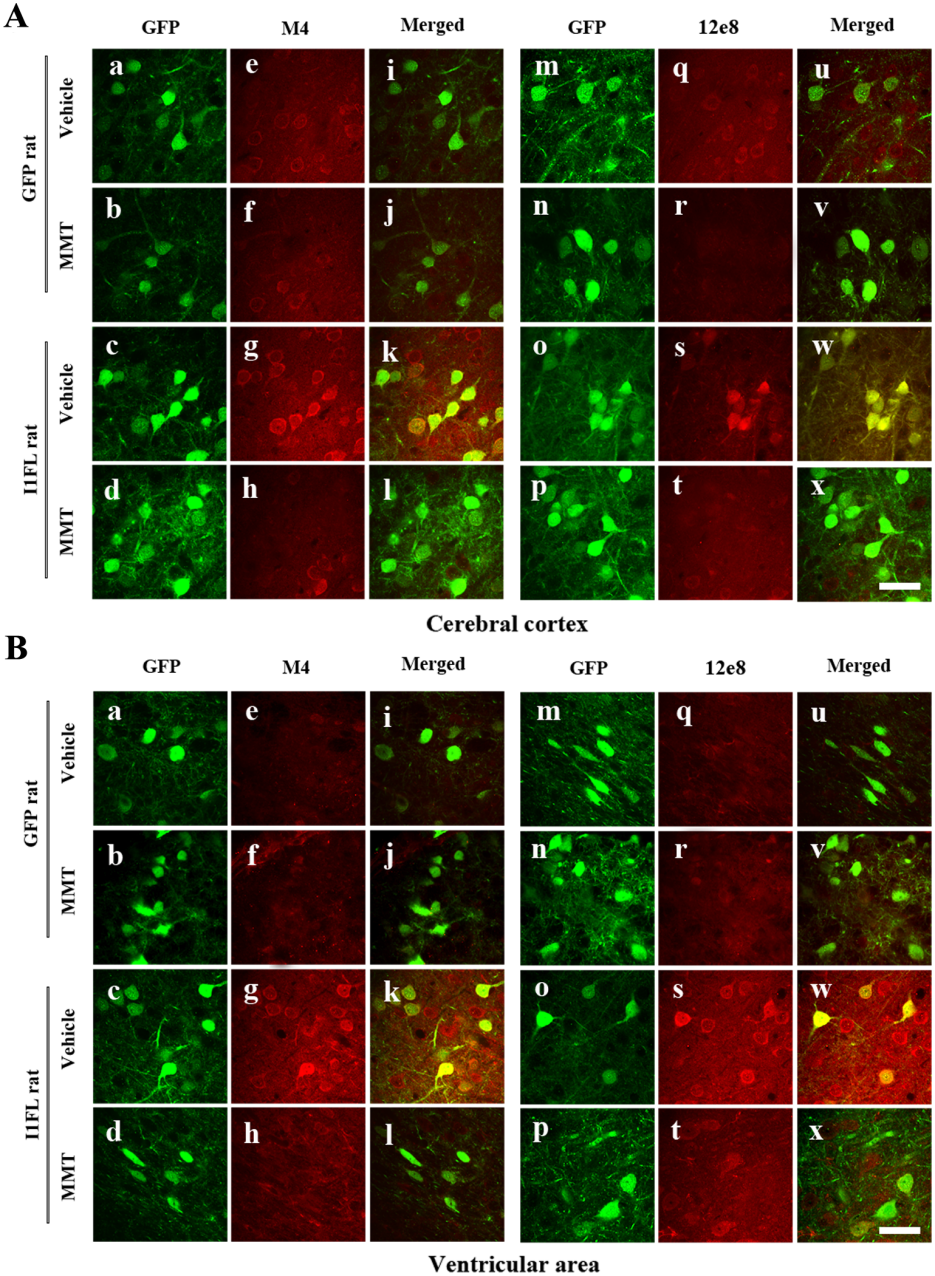
I_1_^PP2A^ and hyperphosphorylated tau are colocalized and memantine reduces expression of hyperphosphorylated tau. A,B) GFP served as a reporter protein for AAV1-induced expression. Strong immunofluorescence in the cerebral cortex (Aa-Ad and Am-Ap), the ventricular area (Ba-Bd and Bm-Bp) was observed. Tau was hyperphosphorylated at pT231/pS235 (M4), and pS262/pS356 (12e8) epitopes in the cerebral cortex (Ag, As), the ventricular area (Bg, Bs) of AAV1-I_1_^PP2A^, but not AAV1-GFP rats (Ae, Aq, Be and Bq). Double immunostaining showed that I_1_^PP2A^ colocalized with hyperphosphorylated tau (Ak, Aw, Bk and Bw). Memantine inhibited tau hyperphosphorylation and phosphorylated tau distribution in the I_1_^PP2A^ rats (Ah, At, Bh and Bt) while memantine didn’t influence the distribution and expression of I_1_^PP2A^ (Ac, Ad, Ao, Ap and Bc, Bd, Bo, Bp). 3–4 rats were employed in each experiment. Scale bar in A and B, 50 μm.

PP2A is the major phosphoserine/phosphothreonine phosphatase that regulates phosphorylation of tau in human brain [4, 33, 34]. Deficiency of PP2A activity either *in vitro* or *in vivo* can lead to abnormal hyperphosphorylation of tau [22, 34]. In the present study, we examined the effect of I_1_^PP2A^ expression on tau phosphorylation in rat brain. We found that tau hyperphosphorylation at Ser-199, Thr205, Thr212, Ser-214, Thr231/Ser-235, Ser-262/Ser-356, Ser-396 and Ser-422 in the ventricular area, the cerebellum, the cerebral cortex and the hippocampus of AAV1-I_1_^PP2A^ rats, was markedly increased when compared with AAV1-GFP control animals (Fig 4A and 4B). Furthermore, we detected the distribution and expression of I_1_^PP2A^ and tau phosphorylation by double immunofluorescent staining in the brain. We observed strong GFP immunofluorescence in the cerebral cortex (Fig 5Aa–5Ad and 5Am–5Ap), the ventricular area (Fig 5Ba–5Bd and 5Bm–5Bp), the hippocampus (not shown) and cerebellum (not shown). These data showed that transgenes were expressed after viral infection and concomitantly tau was abnormally phosphorylated at pT231/pS235 (M4), and pS262/pS356 (12E8) sites in the cerebral cortex (Fig 5Ag and 5As), the ventricular area (Fig 5Bg and 5Bs), the hippocampus (not shown) and cerebellum (not shown) of AAV1-I_1_^PP2A^, but not AAV1-GFP rats (Fig 5Ae, 5Aq, 5Be and 5Bq). Double immunofluorescent staining showed that I_1_^PP2A^ colocalized with hyperphosphorylated tau (Fig 5Ak, 5Aw, 5Bk and 5Bw). These data further supported that the abnormal hyperphosphorylation of tau was most likely due to the inhibition of PP2A activity associated with upregulation of I_1_^PP2A^.

Memantine treatment markedly inhibited tau hyperphosphorylation except some tau sites, which showed a trend of decrease (Fig 4A and 4B). The immunofluorescence staining showed that memantine didn’t influence the distribution and expression of I_1_^PP2A^ (Fig 5Ac, 5Ad, 5Ao, 5Ap, 5Bc, 5Bd, 5Bo and 5Bp). However, memantine inhibited tau hyperphosphorylation and phosphorylated tau distribution in the I_1_^PP2A^ rats (Fig 5Ah, 5At, 5Bh and 5Bt). These findings are consistent with the reversal of PP2A activity by memantine.

**Fig 6:**
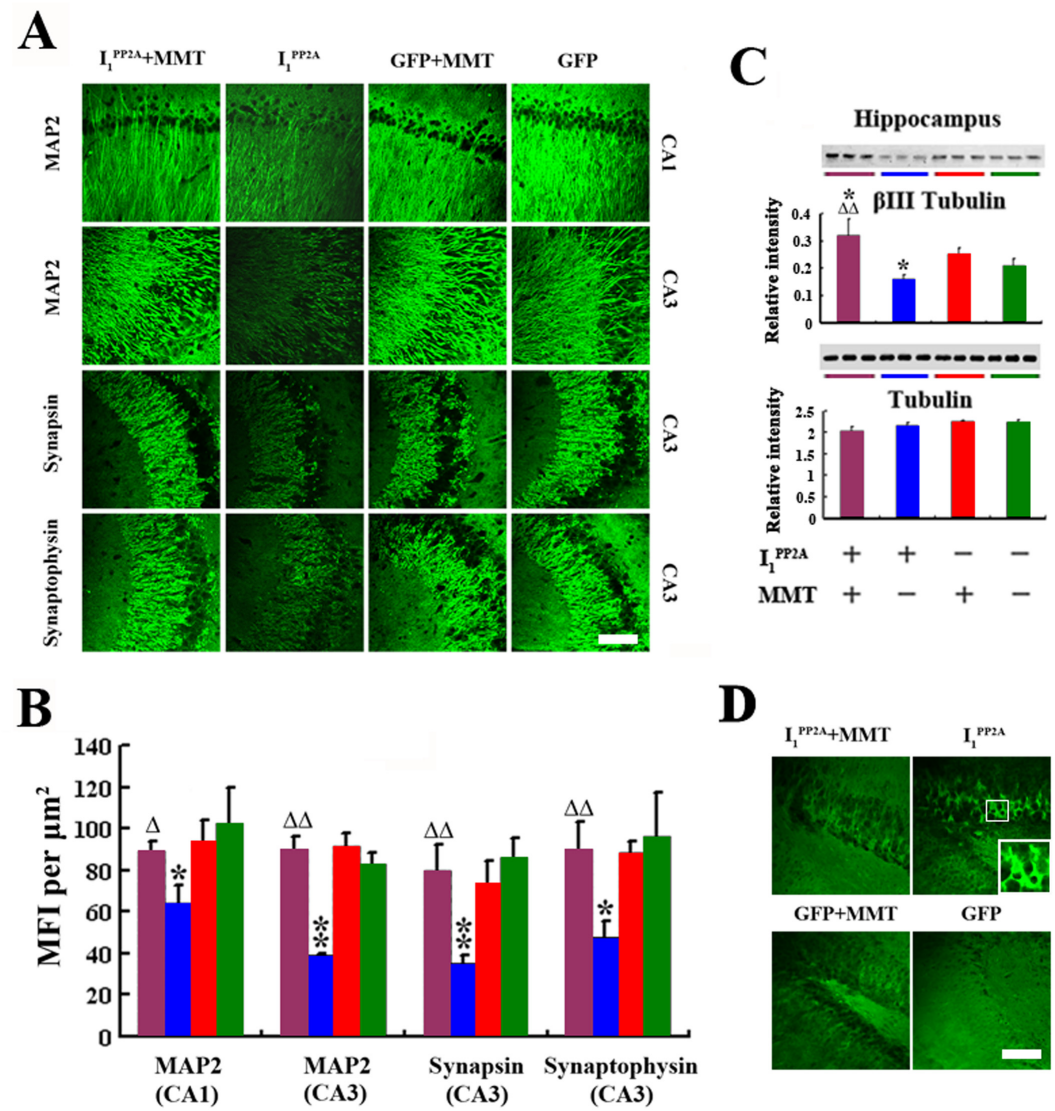
Memantine inhibits I_1_^PP2A^-induced neurodegeneration. A,B) As compared with AAV1-GFP rats, AAV1-I_1_^PP2A^ rats showed a marked decrease in the expression of somatodendritic marker MAP2 and synaptic markers synapsin and synaptophysin in the hippocampus. Memantine treatment restored dendritic and synaptic loss. B) Western blots showed that β III tubulin staining was significantly decreased in the hippocampus of AAV1-I_1_^PP2A^ rats as compared with AAV1-GFP control animals. Memantine restored the decrease to physiological level. C)Fluoro Jade staining showed a selective neurodegeneration in the AAV1-I_1_^PP2A^ rat dentate gyrus of the hippocampus. Memantine prevented the neurodegeneration in the AAV1-I_1_^PP2A^ rats. 3–4 rats were employed in each experiment. *P<0.05, **P<0.01 AAV1-I_1_^PP2A^ vs. AAV1-GFP rats; ^Δ^P<0.05, ^ΔΔ^P<0.01 AAV1-I_1_^PP2A^-memantine vs. AAV1-I_1_^PP2A^-vehicle. Scale bar in A and D, 100 μm.

In our previous study, we found that I_2CTF_ induced inhibition of PP2A activity, and the resulting abnormal hyperphosphorylation of tau produced loss of dendritic and synaptic plasticity and neurodegeneration [22]. To understand the effect of PP2A deficiency and abnormally phosphorylated tau due to I_1_^PP2A^ on neuronal integrity, we studied the expression of MAP2, synaptophysin and synapsin to investigate dendritic and synaptic plasticity, respectively. We found that MFIs of MAP2 immunoreactivity in the pyramidal neurons of CA1 and CA3 regions of the hippocampus were decreased in AAV1-I_1_^PP2A^ rats compared with AAV1-GFP controls (Fig 6A and 6B). Similarly, MFIs of synaptophysin and synapsin were significantly decreased in the CA3 subfield of the hippocampus in AAV1-I_1_^PP2A^ compared with AAV1-GFP rats. We also examined the level of the neuron-specific microtubule protein β III in hippocampus. Western blots results showed that β III tubulin staining was significantly decreased in the hippocampus of AAV1-I_1_^PP2A^ rats compared with AAV1-GFP control animals. The expression of total tubulin, however, was not changed in AAV1-I_1_^PP2A^ rats with or without memantine (Fig 6C). To further investigate neurodegeneration, a sensitive and reliable marker of neuronal degeneration, Fluoro-Jade B staining was employed. We found a significant Fluoro-Jade B staining in dentate gyrus of the hippocampus in AAV1-I_1_^PP2A^, but not AAV1-GFP rats (Fig 6D). Collectively these data suggested that upregulation of I_1_^PP2A^ resulted in dendritic and synaptic loss and neurodegeneration.

### Memantine decreases level of activated GSK-3β and expression of intraneuronal Aβ

**Fig 7:**
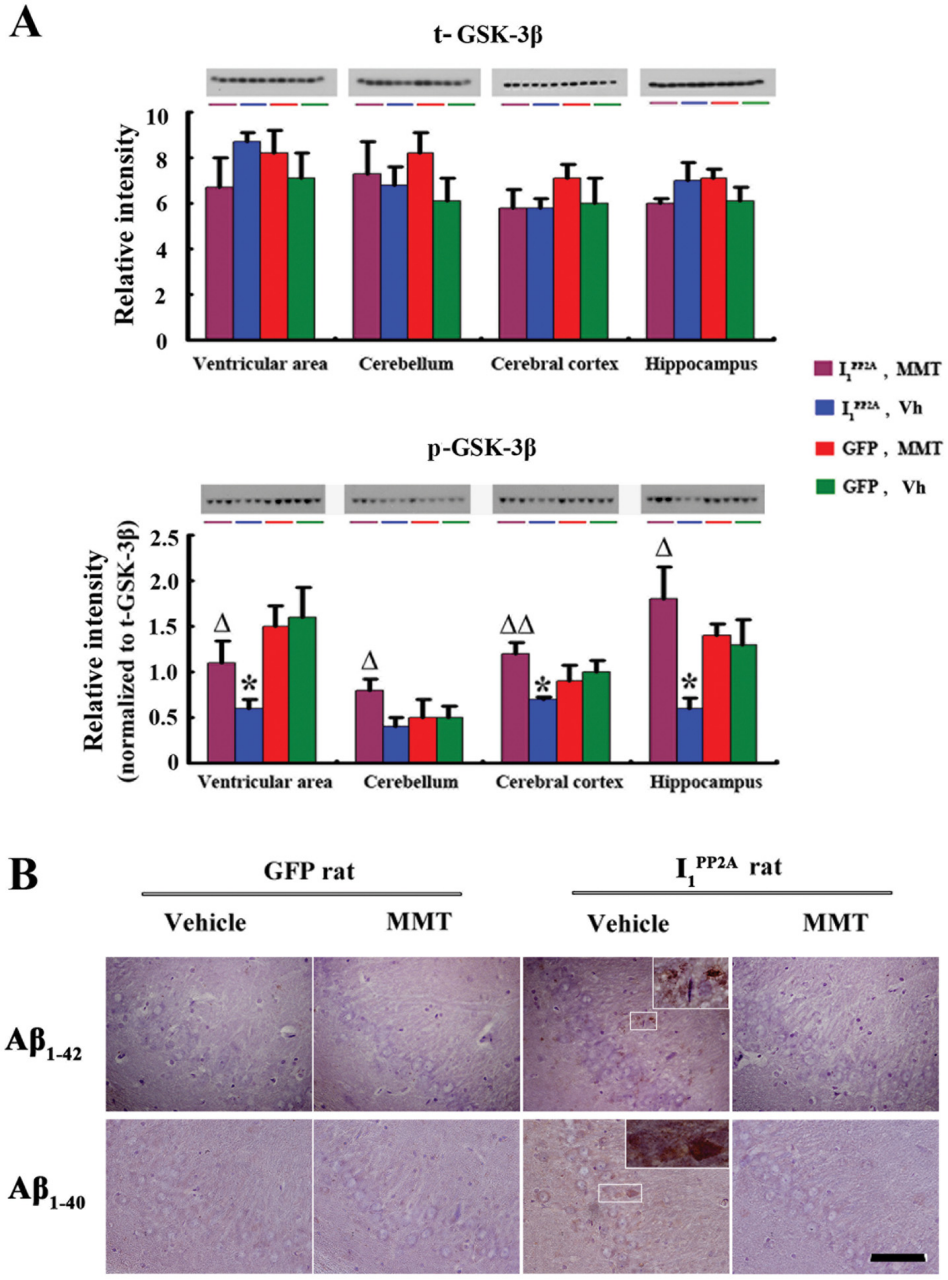
Memantine decreases the level of activated GSK-3β and the expression of intraneuronal Aβ. A) While the level of total GSK-3β was not changed, pSer-9 GSK-3β was markedly decreased in AAV1-I_1_^PP2A^ as compared with the AAV1-GFP rats in hippocampus, cerebral cortex and ventricular areas. Memantine inhibited the phosphorylation of GSK-3β at Ser-9. *P<0.05 vs. AAV1-GFP control rats; ^Δ^P<0.05, ^ΔΔ^P<0.01 vs. AAV1-I_1_^PP2A^ rats. B) Immunohistochemical staining with rabbit polyclonal antibodies to Aβ_34–40_ and to Aβ_36–42_ showed increase in the intraneuronal expression of Aβ in AAV1-I_1_^PP2A^ rats and memantine was found to reverse this change. 3–4 rats were employed in each experiment. Scale bar, 100 μm.

I_1_^PP2A^ and I_2_^PP2A^ were previously reported to reciprocally regulate two major mammalian serine/threonine phosphatases, PP1 and PP2A. In the presence of physiological concentrations of Mn^2+^, I_1_^PP2A^ and I_2_^PP2A^ were found to stimulate PP1_C_ activity by 15–20-fold [35]. PP1 dephosphorylates pSer9 GSK-3β, the inactive form [36, 37]. Taking into account that GSK-3β activity is up-regulated and associated with Alzheimer’s neurofibrillary pathology at all Braak stages [38] and promotes the amyloidogenic processing of βAPP [39], we investigated the level of activated GSK-3β in AAV1-I_1_^PP2A^ rats. We found that while the level of total GSK-3β was not changed, pSer-9 GSK-3β was markedly decreased in the ventricular area, the cerebral cortex and the hippocampus in AAV1-I_1_^PP2A^ as compared with the AAV1-GFP rats. The decrease in the level of pSer-9 GSK-3β was also observed in the cerebellum in AAV1-I_1_^PP2A^ rats but there was no significant difference (Fig 7A). These findings suggested that upregulation of I_1_^PP2A^ induced increase in the level of activated GSK-3β. Next, we examined the expression of intraneuronal Aβ and found that there was an increase in AAV1-I_1_^PP2A^ rats (Fig 7B).

Our previous studies showed that memantine restored the OA-induced changes in the activities of PP-2A, CaMKII and PKA to normal levels and had no significant effect on the activities of cdk5 or GSK-3 in the OA-treated rat hippocampal organotypic cultures [12]. Memantine was reported to significantly protect cultured neurons against Aβ-induced toxicity and attenuate the activation of caspase-3 and GSK-3β in Aβ-treated cultured neurons [16]. In the present study, pSer-9 GSK-3β was restored to a normal level only in AAV1-I_1_^PP2A^ rats after a 12 weeks memantine treatment and had no effect in AAV1-GFP rats (Fig 7A). Consistent with reversal of GSK-3β activity to physiological level, memantine also decreased the expression of intraneuronal Aβ in AAV1-I_1_^PP2A^ rats (Fig 7B). These findings are consistent with the suggestion that β-amyloidosis is associated with activation of GSK-3β.

### Rescue of reference memory impairment and decrease in pSer133 CREB by memantine

During the period of treatment with memantine, the general condition of animals was assessed every week by evaluating grooming and physical state. We did not observe any alteration in general physical state including grooming and posture.

**Fig 8:**
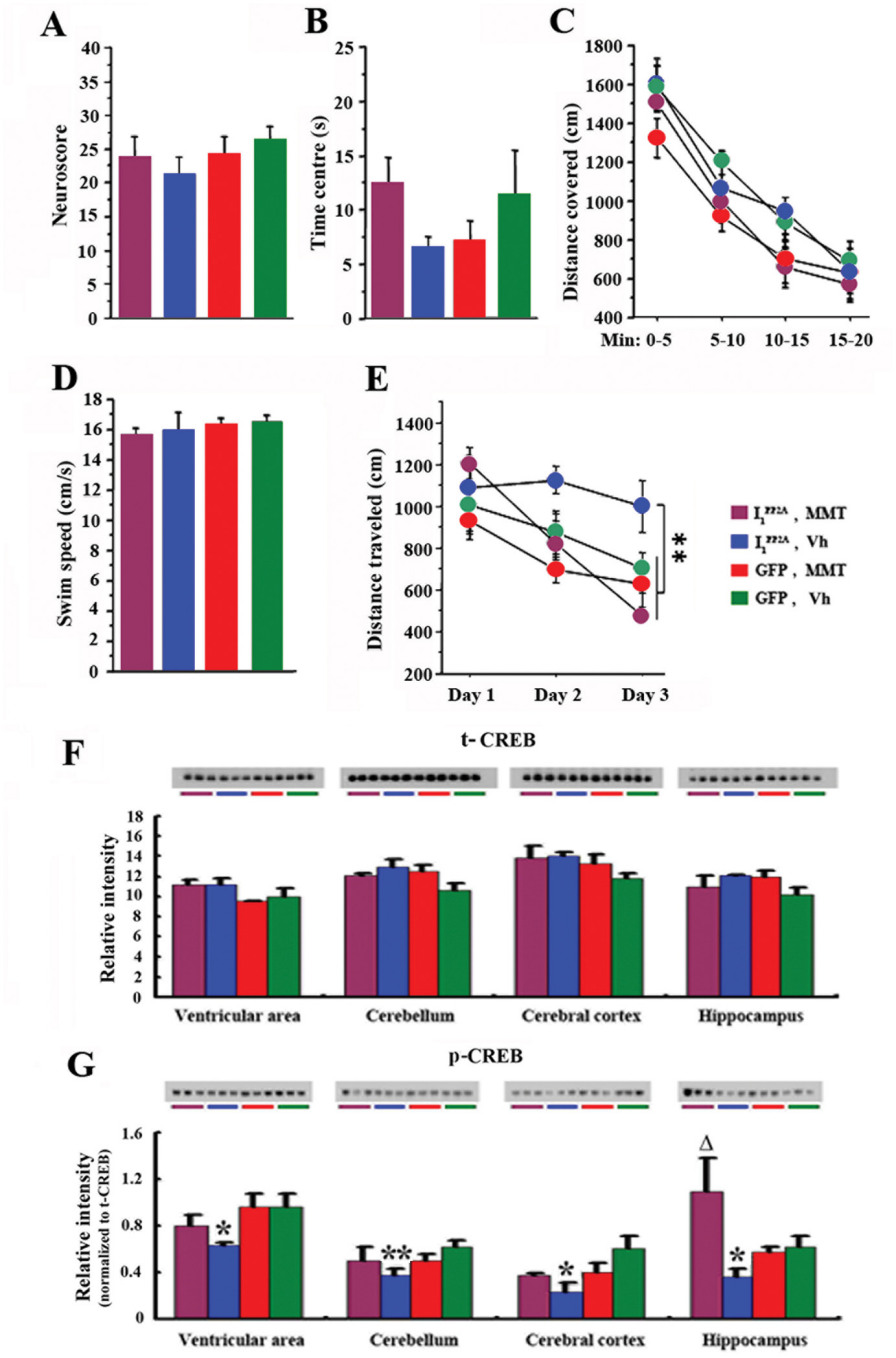
Memantine attenuates spatial learning and memory impairment in AAV1-I_1_^PP2A^ rats. A) Neurological examination did not reveal any significant difference among groups. B) There was no difference between groups to visit the center of the arena in open field, meaning that all animals displayed similar anxiety levels. C) During the twenty minutes of free exploration, all groups covered similar distance, suggesting that all animals exhibited similar level of exploration. D) In the water-maze task, all groups of animals swam at similar speeds. E) During the training of the water-maze task, AAV1-I_1_^PP2A^ rats displayed delayed performance compared to other three groups and the treatment with memantine rescued this impairment. F, G) Western blots data showed that the level of activated phospho-CREB (Ser-133) in AAV1-I_1_^PP2A^ rats was markedly decreased compared with AAV1-GFP control animals in all brain regions studied, while there was no significant change in the level of total CREB. Memantine treatment rescued the phosphorylation of CREB at Ser-133 in hippocampus in AAV1-I_1_^PP2A^ rats. A significant increase of phosphorylation of pSer 133 CREB was found in the ventricular area, the cerebral cortex and the cerebellum in AAV1-I_1_^PP2A^ rats after the memantine treatment. 8–10 rats were employed for behavioral tests. 3–4 rats were employed for Western blots. *P<0.05, **P<0.01 AAV1-I_1_^PP2A^ vs. AAV1-GFP control rats; ^Δ^P<0.05 AAV1-I_1_^PP2A^-memantine vs. AAV1-I_1_^PP2A^-vehicle rats.

Fig 8A represents the neuroscore. Statistical analysis of data obtained from the assessment of neurological examination did not reveal any significant difference among groups (Kruskal-Wallis test, p = 0.507). These results indicate that, neither transduction with AAV1-I_1_^PP2A^ nor memantine treatment induced any changes in neurological function of rats.

There was no difference between the two groups in time spent in the center of the arena (Student t-tests, p>0.050), meaning that all animals displayed similar anxiety levels (Fig 8B).

During the 20 min of free exploration, all groups covered similar distance (Fig 8C, ANOVA, p = 0.259), suggesting that all animals exhibited similar level of exploration.

In the water-maze task, we first analyzed the swim speed of the animals (Fig 8D). Statistical analysis revealed that all rats swam at similar speeds (Student t-tests, p>0.187). Results of the training were then analyzed as distance covered to reach the submerged platform (Fig 8E). During the training of the task, AAV1-I_1_^PP2A^ rats displayed delayed performance compared to other groups (ANOVA, p<0.001; Fisher’s post-hoc test, p<0.022). However, AAV1-I_1_^PP2A^ rats treated with memantine demonstrated training performances similar to control groups (Fisher’s post-hoc test, p>0.333) and more interestingly significantly better than non-treated AAV1-I_1_^PP2A^ rats (Fisher’s post-hoc test, p = 0.012). This finding showed that AAV1-I_1_^PP2A^ rats were impaired in the spatial reference memory task, but that treatment with memantine rescued this impairment.

In order to understand the mechanism of the spatial learning deficit in AAV1-I_1_^PP2A^ rats, we examined the level of CREB, a transcription factor that has been shown to be integral in the formation of spatial memory [40]. We found that the level of activated phospho-CREB (Ser-133) in AAV1-I_1_^PP2A^ rats was markedly decreased compared with AAV1-GFP control animals in all brain regions studied (Fig 8G), while total CREB level showed no significant change (Fig 8F).

## Discussion

Currently, more than 5 million Americans and around 100 million people worldwide suffer from AD, and, barring a medical breakthrough, these numbers are expected to triple by 2050. Today, medical and nursing home bills for AD in the United States alone total about $180 billion a year, a cost expected to reach $1 trillion. These costs will probably increase with the aging of society, becoming a major social problem. Due to a lack of understanding of the exact etiopathogenic mechanisms of this multifactorial disease, its effective therapeutic treatment to date has not materialized. Meanwhile, the sporadic AD accounts for over 99% of the cases [41]. Therefore, a disease-relevant nontransgenic animal model based on the etiopathogenic mechanisms of AD becomes an urgent priority for testing various therapeutic strategies and drug screening.

Previously, we reported a novel etiopathogenic mechanism of AD in which the generation of I_2CTF_ by the cleavage of I_2_^PP2A^/SET initiates a cascade of events, a key feature of which is the inhibition of PP2A activity and resulting AD-like pathologic alterations, including hyperphosphorylation of tau, β-amyloidosis, neurodegeneration and learning and memory impairment [22]. However, I_2_^PP2A^ and I_1_^PP2A^ are two specific endogenous inhibitors of PP2A [42], which both are increased in AD patient brains [9, 10]. Thus, understanding how I_1_^PP2A^ influences PP2A activity and downstream events in AD will help elucidate various etiopathogenic mechanisms involving PP2A deficit.

In the present study, we found that upregulation of I_1_^PP2A^ decreased PP2A activity 4.5 month post-injection of AAV1-I_1_^PP2A^ into newborn rats. The inhibition of PP2A activity led to tau hyperphosphorylation, activation of GSK-3β, increased levels of Aβ, neurodegeneration and learning and memory impairment in AAV1-I_1_^PP2A^ rats. These findings suggest that I_1_^PP2A^ can offer a nontransgenic animal model of AD. Meanwhile, it implies that I_1_^PP2A^ and I_2_^PP2A^ may be jointly considered as molecular targets in AD drug development. That is to say, only blocking one of them might not totally prevent this disease when both I_1_^PP2A^ and I_2_^PP2A^ are increased in the brain.

Memantine is the first in a novel class of AD medications that act on the glutamatergic system by blocking NMDA glutamate receptors [43]. Some studies have shown that memantine can (1) decrease the inhibition of PP2A activity by I_2_^PP2A^ in PC12 cells [19]; (2) inhibit hyperphosphorylation of tau and associated neurodegeneration in hippocampal slices [12]; (3) protect rat cortical cultured neurons against Aβ-induced toxicity [16, 44]; and (4) significantly improve cognitive functions in some AD patients [45, 46]. However, the exact protective mechanism of memantine has not been clear to date. Using AAV1-I_1_^PP2A^ rats, which represent a disease-relevant nontransgenic animal model of AD, in the present study we tested and evaluated the beneficial effect of memantine.

We found that memantine reversed the AAV1-I_1_^PP2A^-induced PP2A inhibition in rats. It is consistent with our previous data (1) that memantine decreases the inhibition of PP2A activity by I_2_^PP2A^ *in vitro* [12]; and (2) that memantine rescues the OA-induced decrease in PP2A activity in PC12 cells [19]. However, less is known about how memantine influences PP2A activity in pathological condition. Therefore, we investigated how memantine regulates PP2A activity in AAV1-I_1_^PP2A^ rats. As a non-competitive NMDA-receptor antagonist with antioxidative properties, memantine interacts with P450 system and inhibits cytochrome P450 mediated monooxygenase functions [30]. Cytochrome P450 monooxygenase possesses the function of demethylation [31]. Meanwhile, methylation of PP2Ac at L309 is an important way to regulate PP2A activity [47]. In the present study, we found that the demethylation of PP2Ac at Leu 309, which reduces its activity, was markedly increased in AAV1-I_1_^PP2A^ rats as compared with AAV1-GFP rats, and memantine restored it to normal level. These findings suggest that inhibition of PP2A with I_1_^PP2A^ is rescued by memantine, decreasing the demethylation of PP2A at Leu 309. Interestingly, we found that memantine did not influence demethylation of PP2A at Leu 309 in AAV1-GFP rats, suggesting that the effect of memantine is primarily by its interaction with I_1_^PP2A^ and not with PP2Ac. This is consistent with our previous findings on the effect of memantine on PP2A activity in I_2_^PP2A^ transfected cells [19].

**Fig 9:**
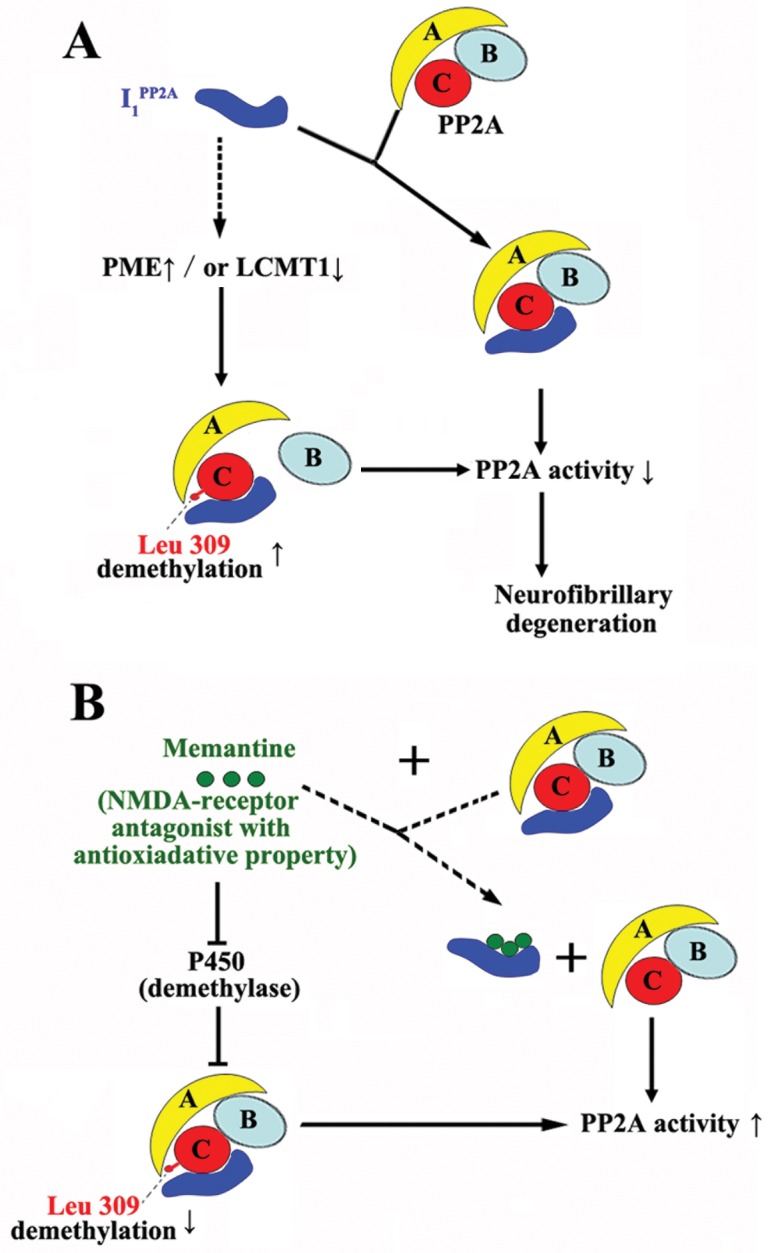
Hypothetical scheme showing how I_1_^PP2A^ inhibits PP2A activity and memantine rescues it. A) I_1_^PP2A^ on the one hand interacts with the catalytic subunit of PP2A (PP2Ac) and inhibits the PP2A activity [8]. On the other hand, I_1_^PP2A^ probably upregulates the methylesterase PME, and/or downregulates the leucine carboxyl methyltransferase LCMT1 (broken line) and leads to an increase in demethylation of PP2Ac at Leu 309, resulting in the decrease of PP2A activity [22]. Demethylation of Leu 309 causes B subunit of PP2A to dissociate from C subunit of PP2A, further decreasing the PP2A activity. B) On the one hand, memantine may competitively bind to I_1_^PP2A^ and rescue PP2A activity (broken line). On the other hand, as a non-competitive NMDA receptor antagonist with anti-oxidative property, memantine may inhibit the cytochrome P450, a demethylase and thereby decrease the demethylation of PP2A at Leu 309 and also lead to rescue of PP2A activity.

Previously, we found that I_1_^PP2A^ interacts with PP2Ac and decreases its activity [8]. We speculate that Leu 309 of PP2Ac is exposed and becomes easy to be demethylated after I_1_^PP2A^ interacts with PP2Ac. Memantine interaction with I_1_^PP2A^ may prevent the exposure of Leu 309 and its demethylation (Fig 9). These findings imply that memantine only restores the pathological change of PP2A to physiological level by decreasing the I_1_^PP2A^-promoted demethylation of PP2A at Leu 309. *In vitro* effect of memantine on PP2A further indicates that it may prevent the decrease of PP2A activity by directly interacting with I_1_^PP2A^, and blocking its binding to PP2A.

Next, due to restoration of PP2A activity, we expectedly found that memantine prevented hyperphosphorylation of tau in AAV1-I_1_^PP2A^ rats. PP2A activity, which accounts for ~70% of the total tau phosphatase in the human brain [4] is compromised in AD brain [5, 6]. Inhibition of PP2A also indirectly promotes the activities of several tau kinases in brain, such as CaMKinase II, PKA, MEK1/2, ERK1/2, and P70S6 kinase [12, 48–50]. Therefore, while memantine restores PP2A activity, it may also attenuate the activities of above-mentioned protein kinases and further result in a decrease in the phosphorylation of tau. In the present study, we found that upregulation of I_1_^PP2A^ induced an increase in the level of activated GSK-3β, and memantine treatment decreased the the level of activated GSK-3β in AAV1-I_1_^PP2A^ rats. PP2A inhibition has been previously shown to lead to GSK-3β inhibition by increasing pSer9 [51–53]. In the presence of physiological concentrations of Mn^2+^, I_1_^PP2A^ and I_2_^PP2A^ are known to stimulate PP1 activity by 15–20-fold [35]. PP1 probably dephosphorylates pSer9 GSK-3β, which overrides its phosphorylation at Ser9, and induces an increase of GSK-3β activity [36]. GSK-3β is known to form a complex with presenilin and enhance its γ-secretase activity and consequently promote β-amyloidosis [54]. Thus, inhibition of PP2A and activation of GSK-3β by upregulation of I_1_^PP2A^ in AAV1-I_1_^PP2A^ rats would also be expected, as shown in the present study, to promote not only neurofibrillary degeneration of abnormally hyperphosphorylated tau but also stimulate β-amyloidosis through increased amyloidogenic processing of APP. Some recent studies have shown that Aβ increases the levels of phospho-Tyr^216^GSK-3β and decreases the level of phospho-Ser^9^ GSK-3β via downregulation of the PI-3/Akt kinase-dependent pathway [16]. Therefore, both Aβ and GSK-3β may promote each other. This relation could further aggravate tau phosphorylation and neurodegeneration.

The present study showed that a chronic treatment with memantine can also attenuate neurodegeneration and the expression of intraneuronal Aβ. Consistent with early reports that Aβ-induced neurodegeneration is mediated through activation of the NMDA receptor [55, 56] and Aβ peptides can enhance glutamate-mediated toxicity in cultured neurons, whereas transient inactivation of the NMDA receptor by antagonists can protect neurons from Aβ toxicity [57–59].

We did not observe any modification of general behavior in AAV-I_1_^PP2A^ rats nor in those treated with memantine. Importantly, this indicated that memantine did not induce any detectable side effects. Regarding cognitive capabilities, we observed that AAV-I_1_^PP2A^ infection induced difficulty to encode, store and/or restitute spatial representation of the environment and coordinates of the submerged platform. These specific impairments are known to reflect hippocampal dysfunctioning [28] and synaptic plasticity failure which is thought to be the cellular substrate of memory [60, 61]. In the present study we observed that memantine treatment could successfully rescue these deficits. It is known that pCREB, an immediate early gene, reflects neuronal activity associated with induction of synaptic plasticity underlying memory consolidation [62]. Therefore, it is legitimate to hypothesize that the rescue of cognitive impairment induced by AAV-I_1_^PP2A^ by memantine could be driven by mechanisms involving CREB/pCREB pathway.

Upregulation of I_1_^PP2A^ may activate PP1, which probably dephosphorylates pSer133 CREB [40, 63] and the decrease of CREB activity probably induces learning and memory impairment. The *in vitro* effect of memantine on I_1_^PP2A^-induced inhibition of PP2A activity in the present study suggests that memantine may directly interact with I_1_^PP2A^, and in turn attenuate the activation of CREB. Accordingly, in the present study learning and memory impairment in AAV1-I_1_^PP2A^ rats was attenuated following a 12 weeks treatment with memantine.

In short, the present study for the first time shows (1) that upregulation of I_1_^PP2A^ induces a decrease in PP2A activity, and results in tau hyperphosphorylation, activation of GSK-3β, increased levels of Aβ, neurodegeneration and learning and memory impairment; (2) that AAV1-I_1_^PP2A^ rats offer a disease-relevant nontransgenic animal model of AD, which recapitulates several key features of the human disease and is relatively simple, rapid, and inexpensive and can be reproduced easily in other laboratories; and (3) that memantine restores the I_1_^PP2A^-induced decrease of PP2A activity to physiological level by decreasing the demethylation of PP2A at Leu 309, and in turn attenuates AD like pathological alteration and behavioral impairment. Thus, the present study provides new insights into the possible mechanism of the therapeutic effect of memantine.

## Supporting Information

S1 File Original data for Fig 3, demethylated-PP2A (dMet-PP2a), for Fig 2D, relative PP2A activity (ELISA normalized by total PP2A (tPP2A) for Fig 2C and tPP2A for Fig 2B. (PDF)
