# Supplementary material for: Memantine Attenuates Alzheimer’s Disease-Like Pathology and Cognitive Impairment
Source: PLoS One. 2015 Dec 23;10(12):e0145441. doi: 10.1371/journal.pone.0145441 (PMC4689401; doi:10.1371/journal.pone.0145441)

S1 File. **Original Data for Figure 3**

PP2A		20 mu/50uL							
I1PP2A	0	5	10	20	40	80	160	320	ng/50uL
	0.221	0.181	0.129	0.11	0.085	0.059	0.052	0.055	
	0.218	0.177	0.142	0.094	0.081	0.066	0.065	0.049	
	0.231	0.188	0.136	0.122	0.088	0.062	0.048	0.048	
x	0.223333	0.182	0.135667	0.108667	0.084667	0.062333	0.055	0.050667	
sd	0.006807	0.005568	0.006506	0.014048	0.003512	0.003512	0.008888	0.003786	

0	5	10	20	40	80	160	320
0.223333	0.182	0.135667	0.108667	0.084667	0.062333	0.055	0.050667

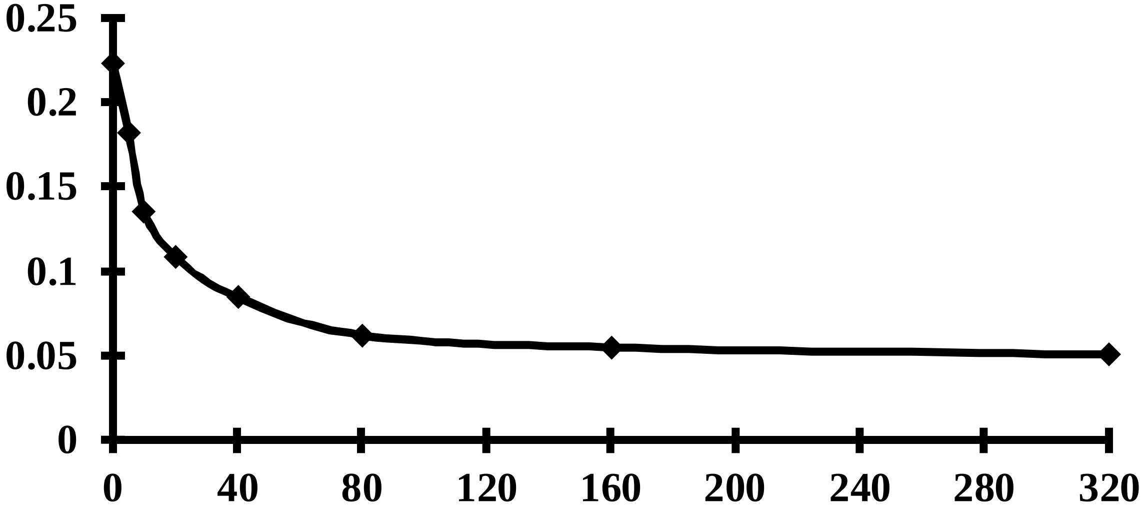

PP2A		20 mu/50uL							
I1PP2A	80					80			ng/50uL

MMT	0	2	5	10	50	100	200	500	1000	ng/50uL
	0.058	0.078	0.082	0.111	0.15	0.223	0.201	0.21	0.219	
	0.06	0.072	0.075	0.102	0.152	0.201	0.22	0.208	0.205	
	0.068	0.068	0.092	0.099	0.178	0.197	0.218	0.223	0.219	
x	0.062	0.072667	0.083	0.104	0.16	0.207	0.213	0.213667	0.214333	
sd	0.005292	0.005033	0.008544	0.006245	0.01562	0.014	0.01044	0.008145	0.008083	

0	2	5	10	50	100	200	500	1000
0.062	0.072667	0.083	0.104	0.16	0.207	0.213	0.213667	0.214333

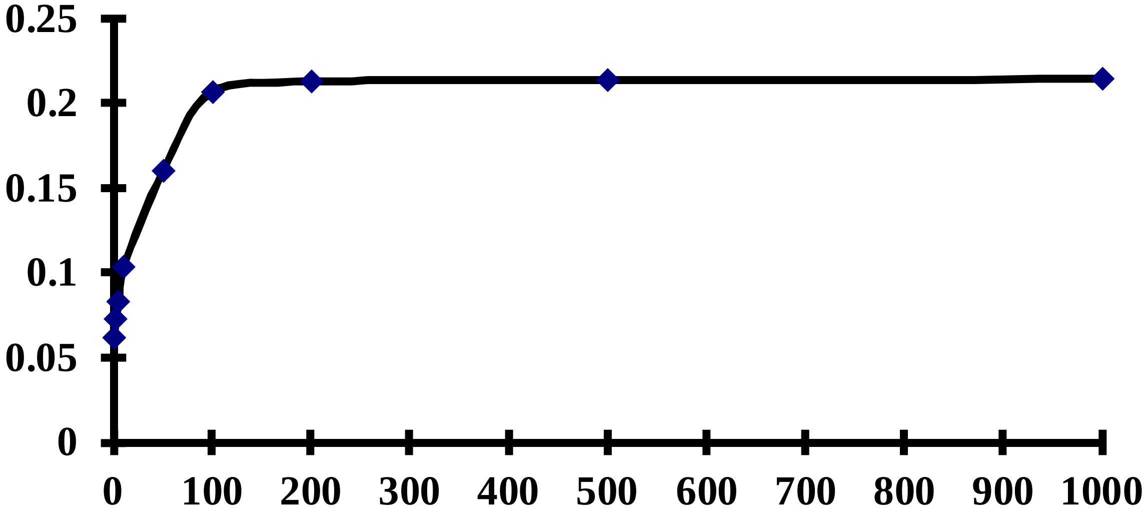

dMet-PP2A-original data for figure 2D

	VR					CB					CC					H			
	I2CTF		GFP			I2CTF		GFP			I2CTF		GFP			I2CTF		GFP	
	MMT	H2O	MMT	H2O		MMT	H2O	MMT	H2O		MMT	H2O	MMT	H2O		MMT	H2O	MMT	H2O
1	58207.48	116954.1	71589.43	68330.16		24398.93	67543.32	43179.26	41541.43	0	50056.56	62524.97	43765.18	36059.13		94462.58	164474.5	77548.17	58020.49
2	57724.32	135617.2	65181.77	61177.35		24320.49	63527.77	51957.87	44033.15		17231.31	85254.59	28911.74	34094.25		85575.45	138471.3	85209.38	42053.48
3	76389.37	144366.1	56243.07	57311.6		36559.6	64687.22	35556.35	39942.99		21868.31	90876.42	23555.06	57161.95		107846.2	146369.8	81524.93	86863.33
tPP2A	477612.2	476975.5	457819.8	527122.2	#	446025.3	373770.0167	489192.3	402011.2	##	522397.2833	490705.41	503878.2	562544.3	##	524452.9	435697.1	463189	480745.4
/tPP2A	0.121872	0.2452	0.156371	0.129629	#	0.054703	0.180708243	0.088266	0.103334	0	0.095820918	0.127418653	0.086857	0.0641	##	0.180117	0.377497	0.160492	0.120689
	0.12086	0.284328	0.142375	0.116059	#	0.054527	0.169964871	0.106212	0.109532	##	0.032985086	0.173738988	0.057378	0.060607	##	0.163171	0.317816	0.183962	0.087476
	0.15994	0.30267	0.12285	0.108725	#	0.081968	0.173066913	0.072684	0.099358	##	0.041861477	0.185195627	0.046748	0.101613	##	0.205636	0.335944	0.176008	0.180685
x	0.134224	0.277399	0.140532	0.118138	#	0.063733	0.174580009	0.089054	0.104075	0	0.05688916	0.162117756	0.063661	0.07544	##	0.182975	0.343752	0.173488	0.129616
sd	0.022277	0.029355	0.016836	0.010606	#	0.015792	0.005529205	0.016778	0.005127	0	0.034006748	0.030591411	0.02078	0.022734	##	0.021376	0.030597	0.011936	0.047242
P vs G	0.321909	0.000905	0.123055			0.013604	8.50576E-05	0.21223			0.476079115	0.016974089	0.543918			0.149277	0.002747	0.193895	
	0.00254					0.000329					0.016338505					0.001725			

x	0.134224	0.277399	0.140532	0.118138	#	0.063733	0.174580009	0.089054	0.104075	0	0.05688916	0.162117756	0.063661	0.07544	##	0.182975	0.343752	0.173488	0.129616
sd	0.022277	0.029355	0.016836	0.010606	#	0.015792	0.005529205	0.016778	0.005127	0	0.034006748	0.030591411	0.02078	0.022734	##	0.021376	0.030597	0.011936	0.047242

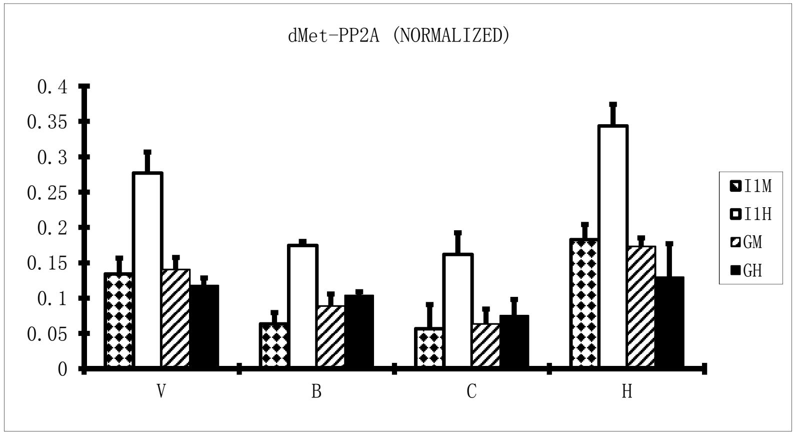

relative PP2A activity (ELISA) normalized by tPP2a-original data for figure 2C

	VR					CB					CC					H			
	I1		GFP			I1		GFP			I1		GFP			I1		GFP	
	MMT	H2O	MMT	H2O		MMT	H2O	MMT	H2O		MMT	H2O	MMT	H2O		MMT	H2O	MMT	H2O
1	0.522	0.279	0.393	0.582		0.515	0.318	0.394	0.418	0	0.633	0.229	0.411	0.469		0.493	0.361	0.57	0.555
2	0.513	0.355	0.535	0.498		0.46	0.366	0.455	0.477		0.48	0.237	0.512	0.52		0.627	0.356	0.62	0.571
3	0.504	0.348	0.573	0.42		0.509	0.287	0.426	0.621		0.421	0.355	0.41	0.585		0.366	0.286	0.444	0.543
pp2a	477612.2	476975.5	457819.8	527122.2	#DIV/0!	446025.3	373770	439192.3	402011.2	5E+05	522397.2833	490705.41	503878.2	562544.3	##	524452.9	435697.1	463189	480745.4
	0.477612	0.476975	0.45782	0.527122	#DIV/0!	0.446025	0.37377	0.439192	0.402011	0.478	0.522397283	0.49070541	0.503878	0.562544	##	0.524453	0.435697	0.463189	0.480745
/PP2A	1.092937	0.584936	0.858416	1.104109	#DIV/0!	1.154644	0.850791	0.897102	1.039773	0.411	1.211722119	0.466680253	0.815674	0.833778	##	0.940122	0.828552	1.230599	1.154566
	1.074094	0.744274	1.168582	0.944753	#DIV/0!	1.031332	0.979212	1.035993	1.186535	0	0.918841418	0.482983493	1.016119	0.924444	##	1.195652	0.817076	1.338546	1.187851
	1.05525	0.729598	1.251584	0.828203	#DIV/0!	1.141192	0.78896	0.969963	1.544734	0	0.805900493	0.723456287	0.813689	1.04	##	0.697941	0.656415	0.958572	1.129603
x	1.074094	0.686269	1.09286	0.959022	#DIV/0!	1.109056	0.872987	0.967686	1.257014	1.074	0.978821343	0.557706678	0.881827	0.932741	##	0.944572	0.767348	1.175906	1.15734
sd	0.018844	0.088063	0.207233	0.138505	#DIV/0!	0.067646	0.097049	0.069474	0.259754	0.019	0.209454032	0.143774645	0.116304	0.103361	##	0.248886	0.096242	0.195803	0.029223
P vs G	0.227047	0.045089	0.405003			0.393772	0.074457	0.135811			0.74976343	0.021420716	0.601171			0.215354	0.002559	0.87884	
	0.001726					0.025903					0.045420968					0.3141			
x	1.074094	0.686269	1.09286	0.948547	#DIV/0!	1.109056	0.865951	0.967686	1.248722	1.074	0.978821343	0.557706678	0.881827	0.932741	##	0.944572	0.767348	1.175906	1.15734
sd	0.018844	0.088063	0.207233	0.1537	#DIV/0!	0.067646	0.106492	0.069474	0.270337	0.019	0.209454032	0.143774645	0.116304	0.103361	##	0.061886	0.096242	0.195803	0.029223

GHH1	GHH2	GHH3	GHC1	GHC2	GHC3
GHB1	GHB2	GHB3	GHV1	GHV2	GHV3
GHMH1	GHMH2	GHMH3	GHMC1	GHMC2	GHMC3
GHMB1	GHMB2	GHMB3	GHMV1	GHMV2	GHMV3
I1HH1	I1HH2	I1HH3	I1HC1	I1HC2	I1HC3
I1HB1	I1HB2	I1HB3	I1HV1	I1HV2	I1HV3
I1HMH1	I1HMH2	I1HMH3	I1HMC1	I1HMC	I1HMC3
I1HMB1	I1HMB2	I1HMB3	I1HMV1	I1HMV	I1HMV3
0.555	0.571	0.543	0.469	0.52	0.585
0.408	0.477	0.621	0.582	0.498	0.42
0.57	0.62	0.444	0.411	0.512	0.41
0.394	0.455	0.426	0.393	0.535	0.573
0.361	0.356	0.286	0.229	0.237	0.355
0.318	0.366	0.287	0.279	0.355	0.348
0.493	0.627	0.366	0.633	0.48	0.421
0.515	0.46	0.509	0.522	0.513	0.504

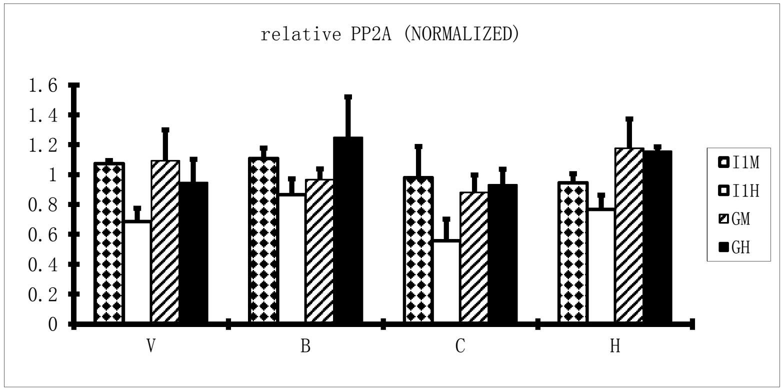

tPP2A -original data for figure 2B

	VR					CB					CC					H			
	I2CTF		GFP			I2CTF		GFP			I2CTF		GFP			I2CTF		GFP	
	MMT	H2O	MMT	H2O		MMT	H2O	MMT	H2O		MMT	H2O	MMT	H2O		MMT	H2O	MMT	H2O
1	519124.9	437685	410559.4	523784.7		478528.4	363499.7	465680.9	404945.1	0	534127.23	497749.4	534316.6	534631.3		582322.9	438851.3	391174.6	470114.9
2	457554.1	485337.4	465963.1	511556.6		432861.3	383165.8	536404.5	360965.9		509236.72	510354.03	510609.5	544078.6		502979.6	465495.6	495845.1	459214.8
3	456157.5	507904.2	496936.8	546025.3		426686.1	374644.6	465491.6	440122.4		523827.9	464012.8	466708.4	608923.1		488056.4	402744.4	502547.2	512906.5
DM1A	205176	210463.3	200881.7	220532.3	#	212515.1	205184.8	199088.3	195222.5	##	151329.3333	167888.3333	153233.7	136392.3	##	205192.4	215862.1	225162.4	223944.8
/DM1A	#REF!	#REF!	#REF!	#REF!	#	#REF!	#REF!	#REF!	#REF!	0	#REF!	#REF!	#REF!	#REF!	##	#REF!	#REF!	#REF!	#REF!
	#REF!	#REF!	#REF!	#REF!	#	#REF!	#REF!	#REF!	#REF!	##	#REF!	#REF!	#REF!	#REF!	##	#REF!	#REF!	#REF!	#REF!
	#REF!	#REF!	#REF!	#REF!	#	#REF!	#REF!	#REF!	#REF!	##	#REF!	#REF!	#REF!	#REF!	##	#REF!	#REF!	#REF!	#REF!
x	477612.2	476975.5	457819.8	527122.2	#	446025.3	373770	489192.3	402011.2	##	522397.2833	490705.41	503878.2	562544.3	##	524452.9	435697.1	463189	480745.4
sd	35957.82	35848.63	43760.7	17475.07	#	28317.35	9862.198	40887.05	39659.72	##	12506.77297	23960.19086	34303.08	40442.02	##	50669.23	31494.26	62456.24	28380.55
P vs G	0.098538	0.094968	0.063481			0.192776	0.297425	0.056926			0.175797666	0.057158804	#REF!			0.262358	0.139526	0.680495	
	#REF!					#REF!					#REF!					#REF!			

x	477612.2	476975.5	457819.8	527122.2	#	446025.3	373770	489192.3	402011.2	##	522397.2833	490705.41	503878.2	562544.3	##	524452.9	435697.1	463189	480745.4
sd	35957.82	35848.63	43760.7	17475.07	#	28317.35	9862.198	40887.05	39659.72	##	12506.77297	23960.19086	34303.08	40442.02	##	50669.23	31494.26	62456.24	28380.55

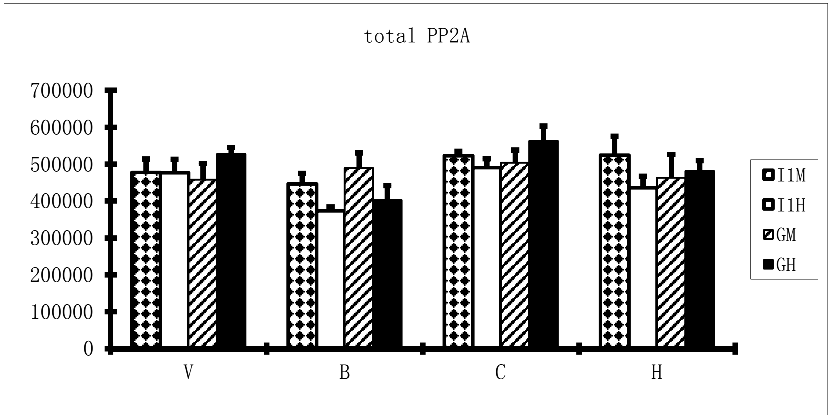

Supplement: S1 File — (PDF) [file pone.0145441.s001.pdf]
